# Engineering *Toxoplasma gondii* secretion systems for intracellular delivery of multiple large therapeutic proteins to neurons

**DOI:** 10.1038/s41564-024-01750-6

**Published:** 2024-07-29

**Authors:** Shahar Bracha, Hannah J. Johnson, Nicole A. Pranckevicius, Francesca Catto, Athena E. Economides, Sergey Litvinov, Karoliina Hassi, Marco Tullio Rigoli, Cristina Cheroni, Matteo Bonfanti, Alessia Valenti, Sarah Stucchi, Shruti Attreya, Paul D. Ross, Daniel Walsh, Nati Malachi, Hagay Livne, Reut Eshel, Vladislav Krupalnik, Doron Levin, Stuart Cobb, Petros Koumoutsakos, Nicolò Caporale, Giuseppe Testa, Adriano Aguzzi, Anita A. Koshy, Lilach Sheiner, Oded Rechavi

**Affiliations:** 1 Department of Neurobiology, Biochemistry and Biophysics, Wise Faculty of Life Sciences and Sagol School for Neuroscience, Tel Aviv University, Tel Aviv, Israel; 2 McGovern Institute for Brain Research, MIT, Cambridge, MA USA; 3 Neuroscience Graduate Interdisciplinary Program, University of Arizona, Tucson, AZ USA; 4 Departments of Neurology and Immunobiology, College of Medicine, and BIO5 Institute, University of Arizona, Tucson, AZ USA; 5 Centre for Parasitology, School of Infection and Immunity, College of Medical, Veterinary and Life Sciences, University of Glasgow, Glasgow, UK; 6 Institute of Neuropathology, University Hospital Zurich, University of Zurich, Zurich, Switzerland; 7 Computational Science and Engineering Laboratory, School of Engineering and Applied Sciences, Harvard University, Cambridge, MA USA; 8 Human Technopole, Milan, Italy; 9 Department of Experimental Oncology, European Institute of Oncology IRCCS, Milan, Italy; 10 Department of Oncology and Hemato-oncology, University of Milan, Milan, Italy; 11 Undergraduate Biology Research Program, University of Arizona, Tucson, AZ USA; 12 Institute of Neuroscience and Psychology, College of Medical, Veterinary and Life Sciences, University of Glasgow, Glasgow, UK; 13 Centre for Discovery Brain Sciences, University of Edinburgh, Edinburgh, UK; 14 Epeius Pharma, Ness Ziona, Israel

**Keywords:** Parasitology, Biologics, Synthetic biology

## Abstract

Delivering macromolecules across biological barriers such as the blood–brain barrier limits their application in vivo. Previous work has demonstrated that *Toxoplasma gondii*, a parasite that naturally travels from the human gut to the central nervous system (CNS), can deliver proteins to host cells. Here we engineered *T. gondii*’s endogenous secretion systems, the rhoptries and dense granules, to deliver multiple large (>100 kDa) therapeutic proteins into neurons via translational fusions to toxofilin and GRA16. We demonstrate delivery in cultured cells, brain organoids and in vivo, and probe protein activity using imaging, pull-down assays, scRNA-seq and fluorescent reporters. We demonstrate robust delivery after intraperitoneal administration in mice and characterize 3D distribution throughout the brain. As proof of concept, we demonstrate GRA16-mediated brain delivery of the MeCP2 protein, a putative therapeutic target for Rett syndrome. By characterizing the potential and current limitations of the system, we aim to guide future improvements that will be required for broader application.

## Abstract (web summary)

*Toxoplasma gondii* secretion systems can be engineered to deliver multiple large therapeutic proteins to neurons in vitro and in vivo.

## Main

Protein delivery requires addressing hard challenges such as bypassing different biological barriers between the site of administration and the target cells, specific tissue targeting and retention of protein integrity until it reaches its target location^1–3^. Proteins are often unstable outside the physiological intracellular environment. This presents a challenge in both the production and delivery of recombinant proteins via oral and intravenous routes (which requires survival of the protein through the digestive and circulatory systems)^1,2^. Free proteins are often immunogenic, which both limits their bioavailable levels and can induce pathological immune reactions^4^. Their large size and often hydrophilic and charged macromolecular nature make them unable to passively pass through biological barriers^1,5,6^. Many proteins require controlled targeting to a specific target tissue, cell type or intracellular compartment for their activity and are ineffective or even deleterious if delivered elsewhere^3^. Particularly challenging is delivery to the central nervous system (CNS), which requires passing through the blood–brain barrier (BBB). The BBB is impermeable to most hydrophilic and large (>400 Da) molecules, which excludes almost all proteins^7^.

Various nanomaterial and protein engineering approaches have attempted to tackle these challenges. Some of these approaches include the use of chemical modifications or molecular fusions to the protein of interest^6,8^, while others include specialized carrier systems made from nanoparticles, liposomes, exosomes and micelles^1,9^. Nevertheless, these approaches tend to have limited efficiency in vivo, often require specific tailoring to each protein of interest, and are applied mostly to peptides or extracellular proteins or binders rather than full-length intracellular proteins^1^. Therefore, a new method that addresses these challenges will have diverse applications in both research and medicine. For research, efficient delivery of protein tools such as antibodies, binders, reporters and genome-editing proteins could facilitate their use in perturbing biological processes in their natural in vivo context. Delivery of natural endogenous or heterologous proteins of interest could enable direct study of their activity, regulation and interactions. For medicine, efficient and safe delivery of proteins could unlock a broad category of protein-based therapies, including endogenous protein replacement therapies for monogenic diseases and heterologous and engineered proteins such as antibody immunotherapies, engineered enzymes, transcription regulators, programmable genetic editors and intracellular signalling peptides^10,11^.

In this paper, we identify *Toxoplasma gondii*, a protozoan brain parasite, as a potential biological tool that can be constructively wielded for delivery of proteins-of-interest to the CNS. *T. gondii* is a ubiquitous parasite that is commonly contracted by ingestion and can actively migrate to the CNS, infiltrating through the BBB via sophisticated mechanisms fine-tuned through their co-evolution with their hosts^12^. In the CNS, *T. gondii* interacts with and persists primarily in neurons^13,14^. *T. gondii* has three organelles for protein secretion: the micronemes, rhoptries and dense granules. Two of these secretion systems can deliver effector proteins directly into host cells. Considering these remarkable innate abilities, we focused our study on delivery of proteins with known utility in neurons. We tested proteins with diverse sizes, functions and intracellular target location, and observed high levels of intracellular delivery for several therapeutic neuronal proteins. We characterized factors affecting delivery as well as the activity of the delivered protein using various in vitro models including neurons and brain organoids. In mice, we show delivery to the brain following intraperitoneal administration and characterize its kinetics. Finally, we use a reporter system to characterize the three-dimensional (3D) distribution of secreted rhoptry and dense granule proteins in the brain. Collectively, our data provide a proof of concept for different applications of engineered *T. gondii* as a vehicle for intracellular protein delivery in vitro and in vivo. We then discuss the potential and limitations of the system and the features that will require further development for future application.

## Results

### Designing protein fusions for *T. gondii*-mediated delivery

Due to their ability to deliver proteins intracellularly, our primary interest was in leveraging the rhoptry and dense granule secretory organelles of *T. gondii*. In the absence of any known trafficking signals to these divergent organelles^15^, we devised a protein fusion strategy. We selected toxofilin (*TGME49_214080*) as a carrier for rhoptry targeting (based on previous fusion studies)^16,17^ and GRA16 (*TGME49_208830*) for dense granule targeting (which we developed in parallel with another project in our group^18,19^). We curated and reviewed a list of all known proteins with demonstrated therapeutic potential for human neurological conditions (Supplementary Table 1) and narrowed it down to our final list of candidates based on a set of detailed guidelines (Extended Data Tables 1 and 2). A subset was tested with both the mammalian and *T. gondii* codon usage (the latter being labelled ‘opt’, for example, *MECP2opt*). In addition, the lysosomal *GALC* gene was expressed both with and without a TAT protein transduction domain which aids cellular cross-correction^20^.

### Targeting heterologous proteins and tools to the rhoptries

**Fig. 1:**
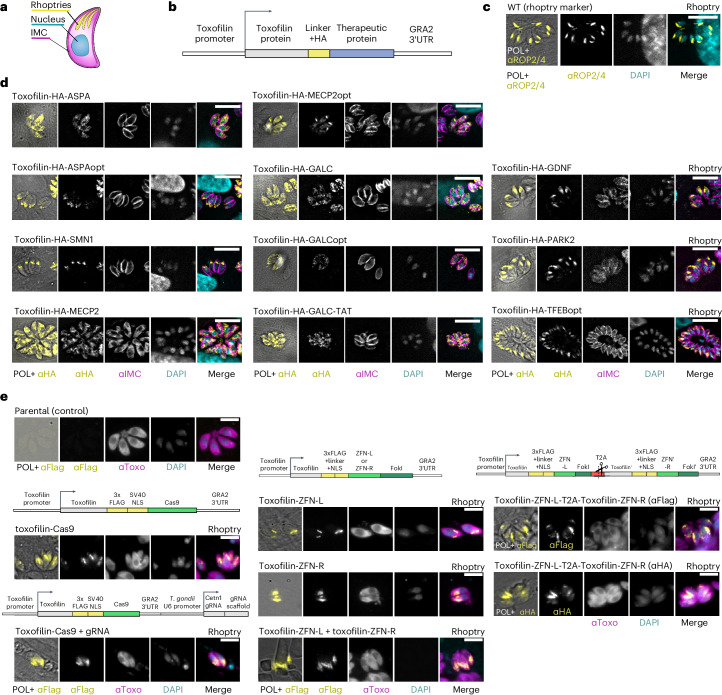
Targeting of therapeutic proteins to the rhoptry secretion organelles by fusion to toxofilin. **a**, Illustration of a *T. gondii* cell. IMC, inner membrane complex (parasite outline). **b**, Scheme of the genetic constructs used. **c**, Intracellular RH *T. gondii* immunostained with the rhoptry marker anti-ROP2/4, in HFF cells. **d**, Intracellular RH *T. gondii* stably expressing different toxofilin-fused therapeutic proteins associated with human neurological diseases, in HFF. **e**, Intracellular *T. gondii* expressing different variations of toxofilin-fused zinc finger nucleases and Cas9. The scheme of each genetic construct is displayed above the image of the *T. gondii* expressing it. The images shown are representative of the protein localization over several independent transfections (numbers of repeats for each are provided in Extended Data Table 2). Scale bars, 10 μm.

To test secretion by the rhoptries, we generated fusions of toxofilin to the neuronal proteins aspartoacylase (*ASPA*, *ASPAopt)*, survival of motor neuron 1 (*SMN1)*, galactosylceramidase (*GALC*, *GALCopt*, *GALC-TAT)*, parkin E3 ubiquitin ligase (*PARK2)*, glial cell derived neurotrophic factor (*GDNF)*, methyl-CpG binding protein 2 (*MECP2*, *MECP2opt)* and transcription factor EB (*TFEBopt)*. Expression was controlled by the predicted endogenous promoter of toxofilin (Fig. 1b). Each fusion protein was assessed with transient (extrachromosomal) and stable (genomically integrated) expression over 2–5 independent transfections. Although many of the tested fusions localized to various unexpected organelles in *T. gondii*, three of the tested fusions, namely, *GDNF*, *PARK2* and *TFEB*, showed successful localization to the rhoptry secretion organelles (Fig. 1d and Extended Data Table 2).

Due to the large dilution factor from the rhoptries to the host cytosol, it was not possible to detect the labelled proteins in the host cells by immunostaining^17^. Faced with this challenge, we were interested in testing delivery of genome-editing proteins, where even a small amount of protein can be detectable by reporter activation (such as in refs. ^16,17^). We tested delivery of engineered Zinc Finger Nucleases (ZFN) and Cas9, which have broad therapeutic potential. Out of five tested variations for ZFN heterodimer and Cas9-gRNA co-delivery, all were successfully expressed and targeted to the rhoptries (Fig. 1e and Extended Data Table 2). However, their activity could not be detected in reporter cells, which can be explained by either low levels of secretion, low nuclease activity, or insufficient timing for reporter induction.

Overall, the correct localization of toxofilin-fused GDNF, Parkin, TFEB, ZFNs and Cas9 to the parasite rhoptries provides a promising step towards using *T. gondii* as a vector for transient invasion-independent delivery but will require further work to increase the levels of secretion and/or activity of the fusion proteins in the host cells.

### Intracellular delivery of proteins via the dense granules

**Fig. 2:**
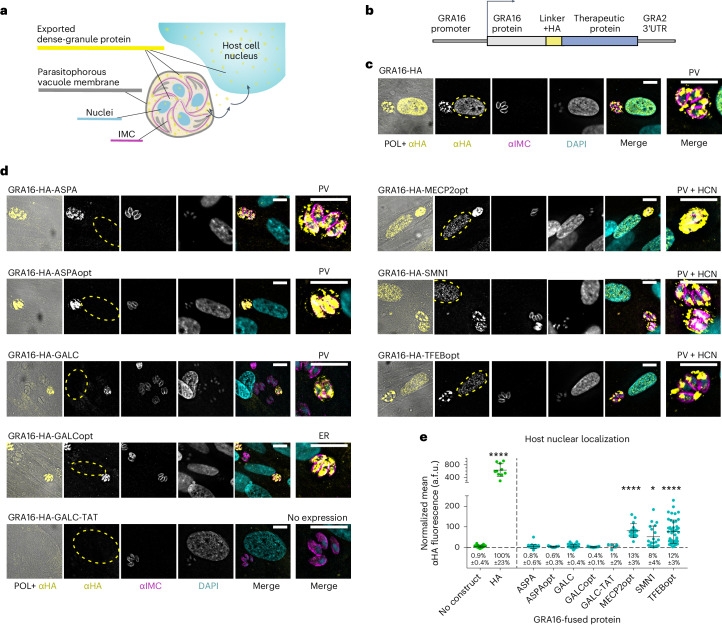
Targeting therapeutic proteins for intracellular delivery by *T. gondii*’s dense granules using fusion to GRA16. **a**, Illustration of an intracellular parasitophorous vacuole (PV) containing four *T. gondii* parasites secreting a dense granule protein (yellow) targeted to the host cell nucleus (HCN). **b**, Scheme of the genetic constructs used. **c**,**d**, Intracellular RH *T. gondii* stably expressing HA-tagged GRA16 alone (**c**) or GRA16-fused proteins associated with human neurological diseases (**d**), in HFF cells. Images represent localization of the proteins to the parasitophorous vacuole and host cell nucleus (*MECP2opt*, *SMN1*, *TFEBopt*), to the parasitophorous vacuole alone (*ASPA, ASPAopt, GALC*), or the most frequently observed localizations in the pool of stably transfected parasites. Rightmost image of each panel shows a close-up view of the parasitophorous vacuole. The numbers of repeated independent transfections for each are provided in Extended Data Table 2. **e**, Fluorescence quantification of the anti-HA signal in the nuclei of infected host cells. Data represent mean ± s.d. *N* = cells per condition, left to right: 32, 10, 30, 11, 39, 23, 6, 17, 20, 47. a.f.u., arbitrary fluorescence units. Significance represents the difference between the fusion protein and the parental strain (‘no construct’), calculated using one-way ANOVA with multiple comparisons (Dunnett test); ****P < 0.0001, *P < 0.0332. Scale bars, 10 μm. Source data

**Extended Data Fig. 1:**
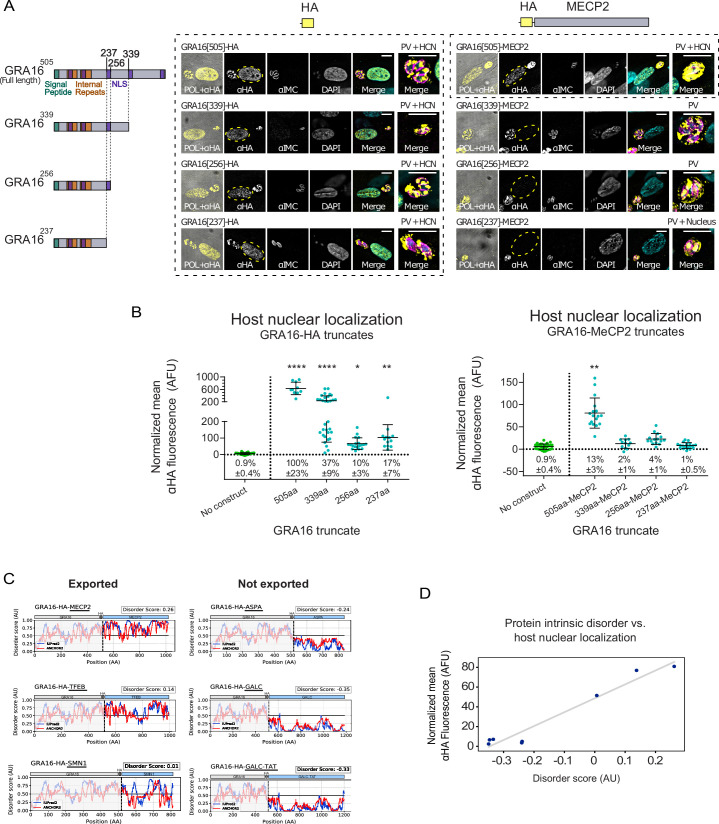
Testing truncated GRA16 for protein delivery and profiling the intrinsic disorder of the tested fusions. (**A**) Intracellular RH T. gondii stably expressing different fragments of GRA16, fused to either a HA tag alone or to the HA-tagged murine MeCP2 (codon optimized). All tagged proteins localized to the PV. However, only the GRA16 truncates without MeCP2 were detected in the host cell nucleus (HCN), as well as the full-length GRA16 with or without MeCP2. Rightmost image of each set shows a close-up of the parasite vacuole, and the text above it indicates the localization of the tagged protein in the image. Yellow dashed lines mark the host cell nucleus. Left and top: illustration of the full-length GRA16, tested C-terminally truncated GRA16, and the fused HA or HA-MECP2 sequences used in the respective constructs. NLS-Nuclear localization signal. (**B**) Fluorescence quantification of the anti-HA signal in the nuclei of infected host cells. Error bars represent mean ± SD. Take note that the y-axis of the leftmost graph is divided into two segments, to account for the high values of GRA16[505]-HA. AFU-arbitrary fluorescence units. Mean relative nuclear localization is normalized to the full-length GRA16[505]-HA. N=cells/condition, left to right: 32,10,32,19,13 (GRA16-HA truncates), 32,17,12,15,18 (GRA16-MeCP2 truncates). Significance represents the difference between the expressed protein and the parental strain (‘no construct’), calculated by two-way ANOVA for the effect of the GRA16 variant and the fused sequence (HA or HA-MECP2) with multiple comparisons (Dunnett test). * P = 0.0332;** P = 0.0021;**** P < 0.0001. Scale bar = 10μm. (**C**) Prediction of protein intrinsic disorder for all tested GRA16 fusion proteins, using IUPred2 (disordered protein regions, blue line) and ANCHOR2 (disordered binding regions, red line). Dashed line marks the start of the heterologous protein, and the grayed-out region before it is GRA16. Disorder score = average (IUPred2 score, ANCHOR2 score) of the fused heterologous protein. (**D**) Correlation between host nuclear localization and the average intrinsic disorder score of the fused heterologous protein. Linear regression line drawn in gray. Source data

To target therapeutic proteins-of-interest to the dense granules, we established a fusion strategy based on the endogenous dense granule protein GRA16. In contrast to rhoptry secretion, which occurs before cell invasion and delivers proteins directly to the host cytosol, proteins secreted from intracellular *T. gondii* via the dense granules are inevitably delivered first to the encapsulating parasitophorous vacuole (PV) that separates intracellular *T. gondii* from the host cytosol (Fig. 2a)^21^. To reach the host cell cytosol, such proteins must be additionally exported through a highly selective protein transport complex on the PV membrane (PVM)^22^. We generated constructs encoding for GRA16 fused to *ASPA*, *ASPAopt*, *SMN1, GALC*, *GALCopt*, *GALC-TAT*, *MECP2*, *MECP2opt* and *TFEBopt*, and used the predicted endogenous promoter of GRA16 (Fig. 2b). *GALCopt* showed localization only inside the *T. gondii*. *GALC-TAT* could not be expressed in *T. gondii* (in four independent transfections). *ASPA*, *ASPAopt* and *GALC* showed protein localization in the PV but not in the host cell, suggesting that the proteins were successfully secreted but remained stuck in the PV. Importantly, the GRA16-fused nuclear proteins SMN1, TFEB and MeCP2 successfully localized to both the PV and host cell nucleus. This indicates that these proteins were successfully targeted to the dense granules, secreted to the PV, exported to the host cell cytosol and accumulated in their site of activity, the host nucleus (Fig. 2d,e and Extended Data Table 2). Notably, these are the full-length mammalian proteins, which together with GRA16 are sized 88 kDa,109 kDa and 110 kDa, respectively. When testing different truncated versions of GRA16, we found that only the full-length GRA16 was able to drive the delivery of a fused protein (Extended Data Fig. 1). Since MeCP2 and TFEB displayed the most robust delivery and targeting, we focused on them for further characterization.

### *T. gondii* can deliver multiple proteins simultaneously

**Fig. 3:**
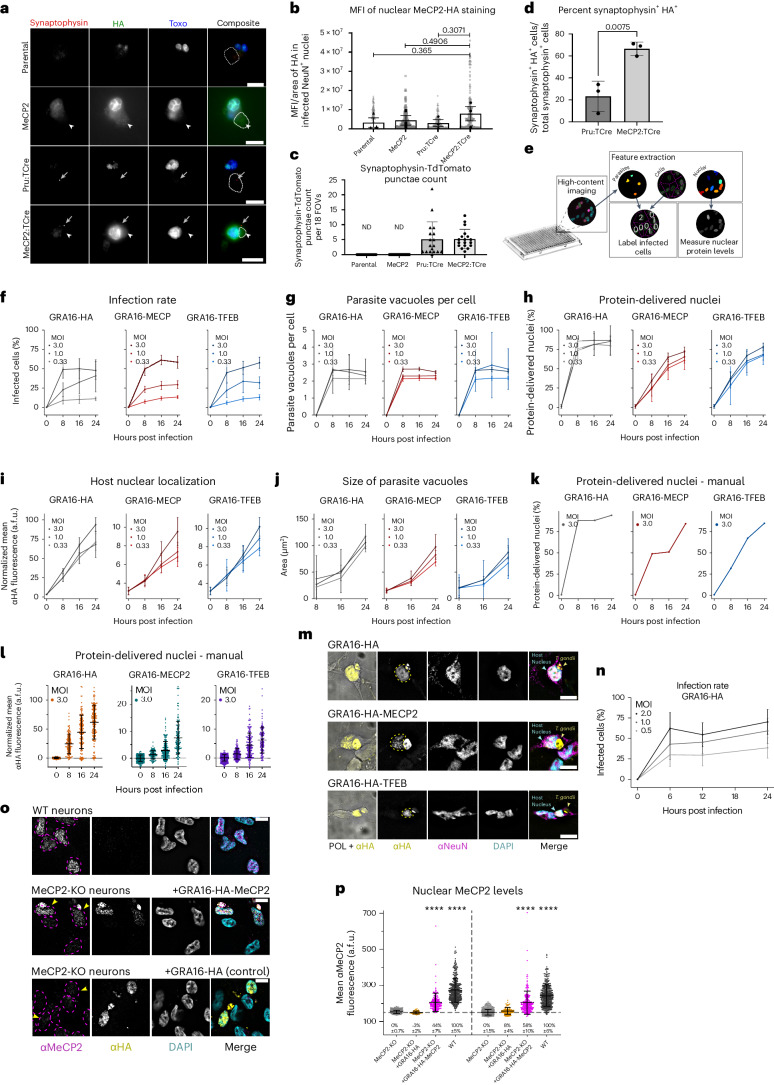
Dual protein secretion, kinetics of dense granule protein delivery and protein delivery to neurons. **a**, Representative images of Synaptophysin-TdTomato neurons infected with *T. gondii* delivering GRA16-MeCP2 alone, toxofilin-Cre (TCre) alone or both. White arrowheads, nuclear GRA16-MeCP2; grey arrows, Synaptophysin-TdTomato punctae from Cre recombination. Scale bars, 20 µM. **b**, Mean fluorescent intensity (MFI) of anti-HA in the nuclei of infected neurons. *N* = 50 images per strain per biological replicate. Black circles, biological replicates; grey circles, technical replicates. **c**, Quantification of Synaptophysin-TdTomato punctae in infected neurons. Symbols, individual FOVs. *N* = 9 FOVs per experiment, 2 independent experiments. ND, not detected. **d**, Quantification of the percent of Synaptophysin^+^HA^+^ neurons of the total Synaptophysin^+^ neurons per strain. *N* = 100 neurons per strain per replicate. Symbols, biological replicates. **b**–**d**, Bars denote mean ± s.e.m., unpaired *t*-test. **e**, Overview of the automated pipeline used for imaging and statistical analysis of *T. gondii*-infected cells. **f**–**l**, Quantitative characterization in infected HFF cells. For all graphs, data represent mean ± s.d. **f**, Infection rate. **g**, PV per infected cell. **h**, Nuclear protein delivery rate (% of infected cells with HA-positive nuclei). **i**, Mean nuclear fluorescence intensity in cells with a single PV. **j**, Increase in PV size demonstrates a doubling time of 7 h for all lines, in agreement with previous reports^120^. **k**,**l**, Manual quantification of protein delivery rate (**k**) and nuclear fluorescence intensity (**l**) for validation of the automated pipeline. *N* = images per condition (*N* for each condition provided in Methods and in Source data). **m**, Representative images of infected LUHMES-derived neurons (3 independent repeats). Yellow dashed lines mark the host nucleus. **n**, Infection rate for GRA16-HA *T. gondii* in LUHMES neurons. Data represent mean ± s.d. *N* = images per condition (*N* for each condition provided in Methods and in Source data). **o**, Uninfected and infected WT and MECP2-KO neurons, 12 hpi. Magenta dashed lines mark neuronal nuclei. Yellow arrowheads mark infected neurons. Scale bars, 10 μm. **p**, Fluorescence quantification of the anti-MeCP2 signal in the nuclei of infected neurons. Data represent mean ± s.d. *N* = cells per condition, left to right: 427, 50, 157, 391, 510, 95, 202, 391. Significance represents the difference between each condition and the untreated MECP2-KO control, two-way ANOVA with multiple comparisons (Dunnett test); ****P < 0.0001. Source data

To test whether the secretion pathways of *T. gondii* can be multiplexed to deliver several proteins by the same vector, we engineered a line for simultaneous delivery from both the rhoptries and dense granules. This line was engineered to express both toxofilin fused to Cre recombinase^16^ and GRA16-MeCP2 (termed MeCP2:TCre). To demonstrate utility as a research tool for studying disease models in vitro, we used cortical and hippocampal primary neuronal cultures from mice with Cre-mediated activation of the Synaptophysin-Tdtomato, a synaptic vesicle protein implicated in X-linked intellectual disability^23^. At 48 h post inoculation, we observed both host cell nuclear accumulation of GRA16-MeCP2 and Synaptophysin-Tdtomato punctae (Fig. 3a–d), indicating dual protein delivery. These results demonstrate that engineered *T. gondii* can deliver proteins from two independent secretion systems concomitantly.

### High-content imaging of dense granule delivery kinetics

We developed a custom high-content imaging pipeline for analysing the kinetics of infection and protein delivery using robotic immunostaining, imaging and quantitative analysis (Fig. 3e). The results showed that 24 h post administration with multiplicity of infection (MOI) = 3, on average 50–62% of human foreskin fibroblast (HFF) cells were infected by *T. gondii* (Fig. 3f,g), and 73–86% of those received the delivered protein (Fig. 3h). *T. gondii* expressing GRA16 alone, GRA16-MeCP2 or GRA16-TFEB were similar in their ability to invade, replicate and deliver proteins to the host cell nuclei (Fig. 3f–h,j). The nuclear levels of the fusion proteins were 8-fold lower than for GRA16 alone (Fig. 3i,l), indicating that while a similar proportion of cells receive the proteins, the quantity of protein delivered is lower. These results provide quantitative characterization of the kinetics of cell infection and protein delivery, and validate a new generalized pipeline for automated high-content imaging and analysis of *T. gondii* protein delivery.

### Delivered MeCP2 binds neuronal chromatin and methylated DNA

To examine *T. gondii*-mediated delivery in neurons, we used human mature dopaminergic neurons differentiated in vitro from Lund Human Mesencephalic (LUHMES) progenitors^24^. Immunofluorescence of neurons inoculated with GRA16-HA, GRA16-MeCP2 and GRA16-TFEB *T. gondii* confirmed that all could efficiently infect and deliver the proteins to neurons (Fig. 3m,n). We then used *MECP2* knockout LUHMES neurons^25^ to quantify the levels of MeCP2 delivery and found that 24 h after inoculation, it reaches 58% of WT MeCP2 levels (Fig. 3o,p). These values are comparable to protein levels achieved in previous studies of *MECP2* reactivation and viral gene therapy for Rett syndrome^26–29^.

**Fig. 4:**
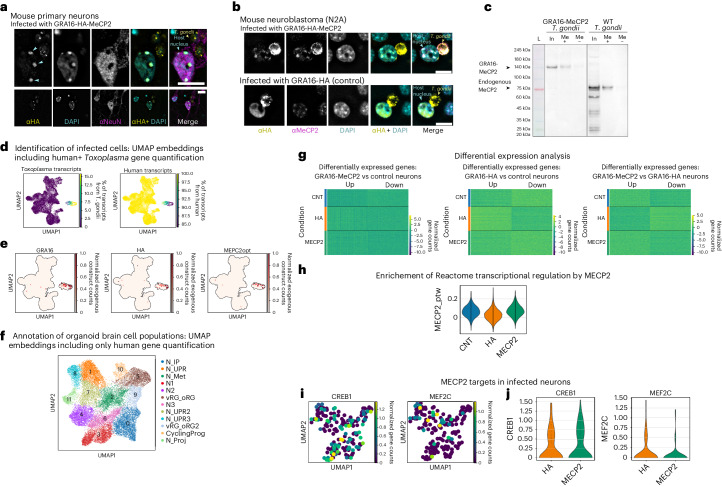
Probing the functionality of *T. gondii*-delivered MeCP2 via heterochromatin binding, pull-down assay and single-cell sequencing in human cortical organoids. **a**, Mouse primary neurons inoculated with GRA16-MeCP2. Top images show a close-up view of the soma and *T. gondii* (2 independent repeats). Blue arrowheads mark co-localization of GRA16-MeCP2 with foci of heterochromatic DNA. Scale bars, 10 μm. **b**, Mouse neuroblastoma cells inoculated with GRA16-MeCP2 and GRA16-HA *T. gondii* (2 independent repeats). Scale bars, 10 μm. **c**, Pull-down assay of protein lysates from HFF cells infected with GRA16-MeCP2 or WT ME49 *T. gondii*. L, protein ladder; In, Input lysate; Me(+), protein pull down with methylated DNA probes; Me(−), protein pull down with non-methylated DNA probes; primary antibody, anti-MeCP2. Representative blot from 3 independent repeats. **d**, UMAP of single cells based on human+*T. gondii* gene quantification, coloured by percentage of *Toxoplasma* and human transcript counts. **e**, Same UMAP as in **d** but coloured by exogenous constructs transcript counts. **f**, UMAP of single cells based on human gene expression. Main clusters were identified and coloured by cell subtypes. CyclingProg, cycling progenitors; vRG_oRG, vRG_oRG2, ventricular and outer radial glia; N_IP, neuronal intermediate progenitors; N1, N2, N3, neuronal clusters; N_UPR, N_UPR2, N_UPR3, neurons with signature of unfolded protein response; N_Met, neurons with signature of metabolic regulation; N_Proj, neurons with signature of axonal regulation. **g**, Heat map showing the distribution of differentially expressed genes between neurons of organoids infected with GRA16-MeCP2 vs uninfected (left),GRA16-HA vs uninfected (middle) and GRA16-MeCP2 vs GRA16-HA organoids (right). **h**, Violin plot showing mean expression of genes belonging to the ‘Reactome transcriptional regulation by MECP2’ pathway (10.3180/R-HSA-8986944.1); norm. enrich. = 3.19, *P* = 0.001417 for the GRA16-MeCP2 vs GRA16-HA comparison. **i**, UMAP of single infected neurons coloured by expression of *CREB1* and *MEF2C*. **j**, Violin plots showing the counts distribution of *CREB1* and *MEF2C* between neurons from organoids infected with GRA16-HA and GRA16-MeCP2. All scRNA-seq data include 3 biological replicates (single-cell suspensions dissociated from 3 different organoids) for each condition. Source data

MeCP2’s ability to specifically bind heterochromatic DNA provides a marker for its functionality^30,31^. Using mouse primary neurons, where the heterochromatin can be readily visualized, we demonstrated that GRA16-MeCP2 co-localizes to foci of heterochromatic DNA in infected neurons (Fig. 4a), mimicking the functional endogenous MeCP2 (ref. ^30^). The same specific binding was observed following treatment of mouse neuroblastoma cells (Fig. 4b).

To further demonstrate the functionality of *T. gondii*-delivered MeCP2, we tested its binding to methylated DNA using a pull-down assay. Protein lysates from cells infected with GRA16-MeCP2 and control *T. gondii* were assayed for binding of methylated and unmethylated DNA. The *T. gondii*-delivered GRA16-MeCP2 demonstrated specific binding of methylated DNA, similar to the endogenous human MeCP2 (Fig. 4c). These results provide preliminary evidence for GRA16-MeCP2’s ability to functionally bind target DNA in neurons.

### MeCP2 delivery alters gene expression in brain organoids

**Extended Data Fig. 2:**
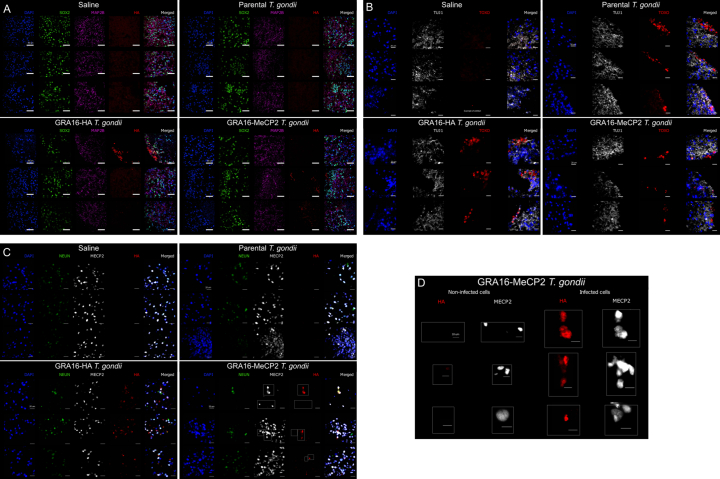
Delivery of proteins to human brain organoids. Representative immunostaining for (**A**) anti-SOX2 (green), anti-MAP2B (pink), anti-HA (red), DAPI (blue). (**B**) anti-TUJ1 (white), anti-Toxo (red), DAPI (blue). (**C**) anti-NeuN (green), anti-MeCP2 (white), anti-HA (red) and DAPI (blue). Scale bars = 20 μm. (**D**) enlarged sections containing infected and uninfected cells from C. Scale bars = 10 μm. N = 3 replicates (organoids) for each strain and combination of antibodies.

**Extended Data Fig. 3:**
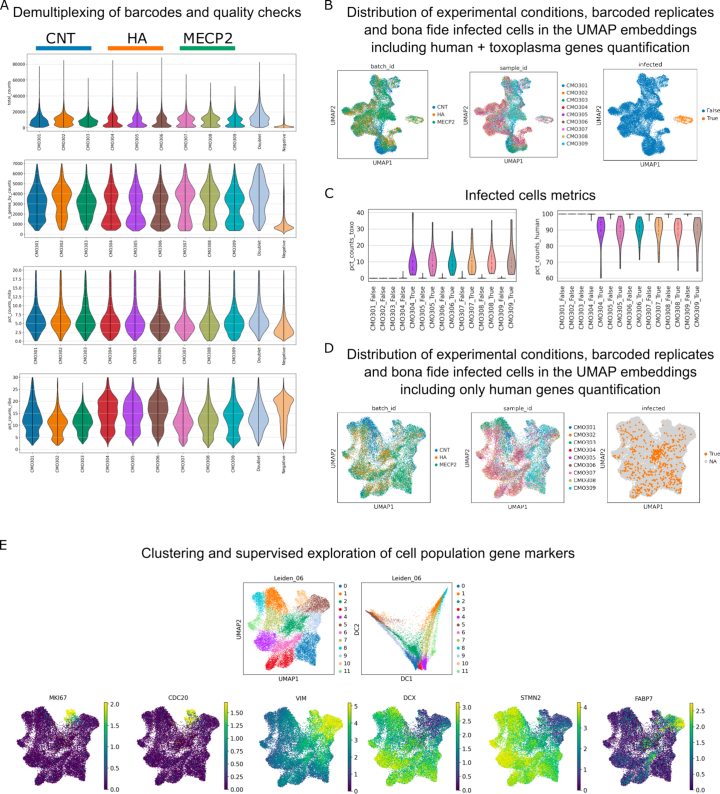
Extended single-cell sequencing analyses including quality checks and normalization, identification of bona fide infected cells, clustering, gene markers and the MECP2 reactome pathway. (**A**) Violin plots showing the distribution of the relevant metrics for quality checks across all the replicates of each infection condition, and the droplets identified as doublets and as empty droplets. All scRNA-seq data includes three biological replicates (single cell suspensions dissociated from 3 different organoids) for each condition. (**B**) UMAP plots of the single cells colored by experimental conditions, barcoded replicates, and bona fide infected cells in the UMAP embeddings including human + T. gondii genes quantification; (**C**) Violin plots showing the distribution of human vs T. gondii transcripts across all replicates, after having identified the infected cells; (**D**) UMAP plots of the single cells colored by experimental conditions, barcoded replicates, and bona fide infected cells in the UMAP embeddings including only human genes quantification; (**E**) UMAP and diffusion map plots of the single-cell colored by Leiden cluster; UMAP plots colored by paradigmatic marker of neurodevelopmental cell types.

To further investigate the function of the delivered MeCP2, we analysed the transcriptional effects of protein delivery in human cortical brain organoids^32,33^. Imaging of late stage (Day 200) organoids demonstrated robust infection and protein delivery to neurons in the organoids (Extended Data Fig. 2). We thus performed single-cell sequencing to obtain transcriptomic profiles of infected and control organoids (Day 50) to study the process at the molecular level. After preprocessing and filtering (Fig. 4d,e and Extended Data Fig. 3), we normalized and integrated data for 57,818 cells, performed dimensionality reduction, clustering and supervised exploration of marker genes to characterize the different neurodevelopmental cell types and subtypes present in our cohort of organoids (Fig. 4f, and Extended Data Fig. 3 and Table 3). Differential expression analysis of the infected and uninfected organoids demonstrated differential modulation of the ‘Reactome transcriptional regulation by MECP2’ pathway in neurons infected with GRA16-MeCP2 *T. gondii* compared with GRA16-HA (Fig. 4g,h and Extended Data Fig. 3). These effects came up as significantly enriched from gene set enrichment analysis of the differentially expressed genes (norm. enrich. = 3.19, *P* = 0.001417). Upon performing a focused analysis of specific MeCP2 targets^34^, we observed modest upregulation of *CREB1* coupled with a downregulation of *MEF2C* (Fig. 4i,j). Collectively, these observations provide additional preliminary support for delivery of functional MeCP2 by the engineered *T. gondii*.

### Brain delivery following systemic administration in mice

**Fig. 5:**
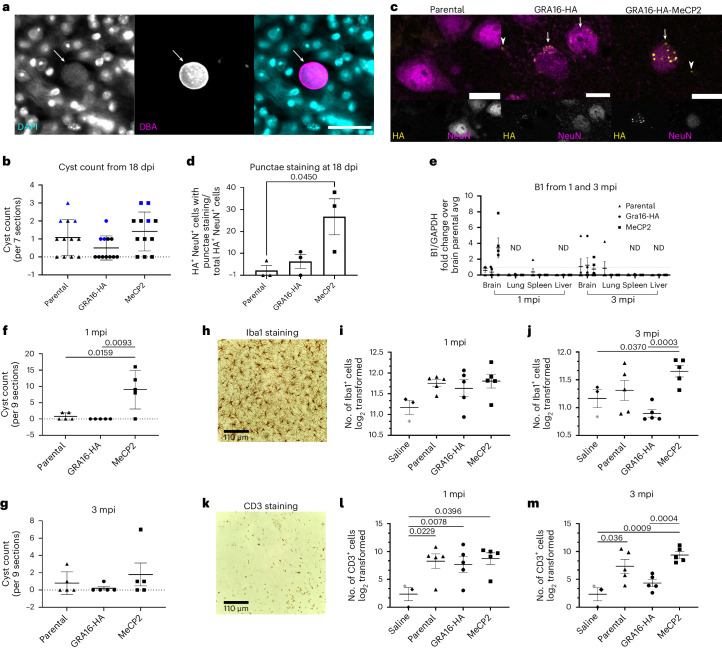
*T. gondii* delivers MeCP2 to the CNS following intraperitoneal administration in mice. Mice were injected intraperitoneally with saline, the parental ‘Pru’ (reduced virulence) strain, GRA16-HA or GRA16-MeCP2 Pru *T. gondii* as labelled. At 18 dpi (**a**–**d**) or 1 and 3 mpi (**e**–**m**), brains were collected and processed for analysis. **a**, Representative maximal projection image of a cyst. Scale bar, 20 µM. **b**, Quantification of cyst numbers per 7 sections per mouse at 18 dpi. *N* = 12 mice per group. Blue symbols represent mice used to quantify punctate HA staining in **c** and **d**. Data represent mean ± s.d. **c**, Representative confocal images from brain sections stained with anti-HA (yellow) and anti-NeuN (magenta) from 18 dpi mice. Arrows point to cells that show co-localization of anti-HA and anti-NeuN staining; arrowheads point to anti-HA staining that did not co-localize with anti-NeuN staining. HA staining for GRA16-HA-infected tissue was typically diffuse while GRA16-MeCP2 was punctate. Scale bar, 10 µM. **d**, Quantification of the HA^+^NeuN^+^ punctate staining as a percentage of all HA^+^NeuN^+^ staining; 150 FOVs per mouse, *N* = 3 mice per group. **e**, Parasite burden in indicated organs by qPCR of the parasite-specific B1, normalized to GAPDH. All results normalized to the average brain burden for parental *T. gondii* at 1 mpi. **f**,**g**, Quantification of cysts per 9 sections per mouse, *N* = 5 mice per group at 1 (**f**) and 3 (**g**) mpi. **h**, Representative image of brain section stained with anti-Iba1 antibodies (microglia and macrophages). Scale bar, 110 µM. **i**,**j**, Number of Iba1^+^ cells per brain section per mouse at 1 (**i**) and 3 (**j**) mpi. *N* = 3 sections per mouse. **k**, Representative image of brain section stained with anti-CD3ε antibody (T cells). Scale bar, 110 µM. **l**,**m**, Number of CD3^+^ cells at 1 (**l**) and 3 (**m**) mpi. *N* = 5 mice per group for the infected mice (1 and 3 mpi); *N* = 3 mice per group for saline. The saline group was combined from the 1 mpi (black diamonds) and 3 mpi (grey diamond) cohorts. For all graphs, data represent mean ± s.e.m.; one-way ANOVA with subsequent two-tailed *t*-tests were used to calculate significance.

Having shown that *T. gondii* can deliver MeCP2 in cultured mammalian cell lines, neurons and brain organoids, we next sought to study protein delivery in vivo. As the lines based on Type I RH *T. gondii* were unable to efficiently convert to the bradyzoite stage, we engineered new lines based on the Type II Pru strain, which has lower virulence in vivo and can establish latent asymptomatic infection in mice^35^. These lines were used to intraperitoneally inoculate mice whose brains were collected 18 days post injection (dpi). All mice displayed high levels of brain bradyzoite cysts regardless of the *T. gondii* line used, although mice injected with the GRA16-HA *T. gondii* displayed slightly lower cyst counts than the others (Fig. 5a,b). Confocal imaging of brain sections from three of the mice that had the highest cyst counts in each group (blue dots in Fig. 5b) demonstrated HA staining in the neuronal nuclei of mice injected with the GRA16-HA or GRA16-MeCP2 *T. gondii* (Fig. 5c,d). Nuclear HA staining displayed two visible types: punctate or diffuse. The punctate localization was enriched in neurons with the GRA16-MeCP2 *T. gondii* (Fig. 5d) and was similar to the localization of the endogenous mouse MeCP2 and of GRA16-MeCP2 in vitro. Overall, these findings demonstrate that *T. gondii* can deliver MeCP2 to neurons in the brain of mice.

**Extended Data Fig. 4:**
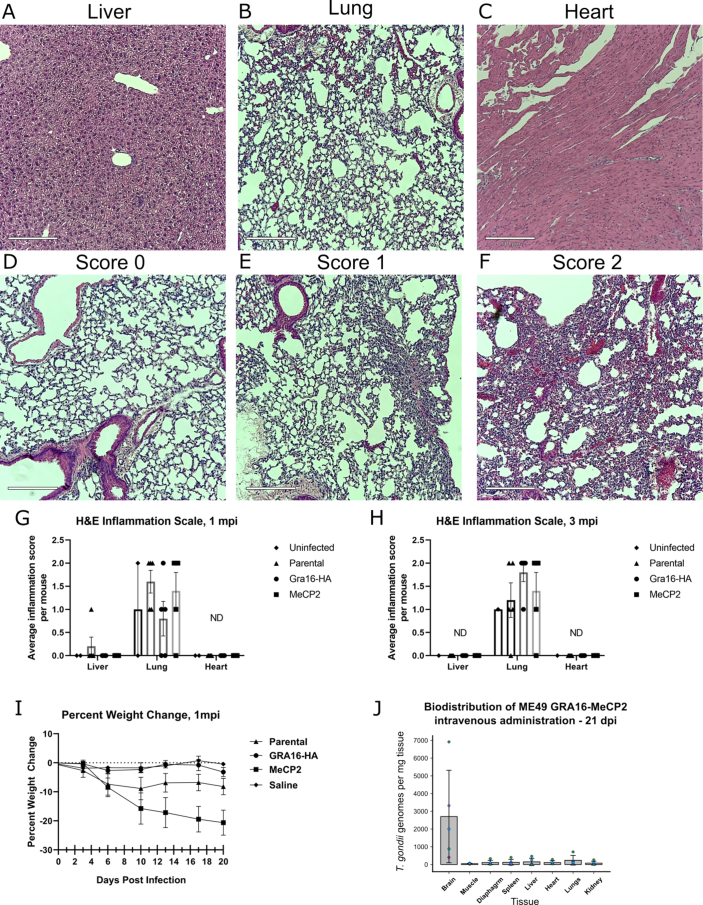
MeCP2 does not alter peripheral inflammation, and brain distribution following intravenous administration. (**A**–**I**) Mice were inoculated with 20,000 freshly lysed Pru (reduced virulence) T. gondii tachyzoites of the identified strains. At labelled time points, organs were harvested, fixed, sectioned, stained, and analyzed. N = 5 mice/group/time point for the infected conditions and N = 2 mice/group for the uninfected controls (independent biological repeats). The uninfected control mice are combined from the two time cohorts (1 from 1 mpi and 1 from 3 mpi). (**A**–**C**) Representative images of formalin-fixed, H&E-stained tissue sections from labelled organs at 1 mpi. Scale bar=210 µM. (**D–F**) Representative images of lung sections that were given inflammation scores of 0, 1, or 2, respectively. Inflammation scoring system: Tissue with no inflammation = 0, some inflammation = 1, and notable inflammation = 2. Scale bar = 210 µM. (**G**–**H**) Graph of average inflammation score for labelled organs at 1 mpi (**G**) and 3 mpi (H). Inflammation score generated from 8 FOV/organ, 1 section/organ/mouse. Bars, mean ± SEM. ND = not detected. (**I**) Percent weight change of infected and saline-inoculated mice during the first 20 days of infection. Mice were weighed before infection or saline-inoculation and throughout infection as indicated. Symbols, mean ± SD. (**J**) Male mice were injected in the tail vein with 50,000 tachyzoites of ME49 GRA16-MeCP2 T. gondii. At 21 days post-injection, the tissues were harvested and DNA was extracted. T. gondii genomes per mg tissue was measured by qPCR of the T. gondii B1 gene. Genome number was calculated from the Ct values based on a standard curve generated from a serial dilution of a B1 plasmid, assuming 35 B1 copies per genome. At 21 dpi, all peripheral tissues have significantly lower amounts of T. gondii than the brain (P < 0.001, one-way ANOVA), N = 5 mice/group. Bars show mean ± std, and each color corresponds to a different mouse.

To test whether the genetic modifications performed (namely, the heterologous expression of MeCP2) affected the kinetics of infection or *T. gondii’s* distribution in the body, we compared total *T. gondii* levels in the brain and in peripheral tissues (liver, lung and spleen) at 1 and 3 months post injection (mpi). In both 1 and 3 mpi, *T. gondii* was primarily detected in the brain, with little *T. gondii* detected in the periphery (Fig. 5e–g). The same results were observed following intravenous administration of ME49 GRA16-MeCP2 (Extended Data Fig. 4j).

Histological markers of peripheral inflammation were similar in mice injected with saline and *T. gondii*, indicating minimal inflammation in the periphery at 1 and 3 mpi (Extended Data Fig. 4). Brain inflammation at 1 and 3 mpi consisted of high levels of infiltrating monocytes (Iba1^+^ cells) and infiltrating T cells (CD3ε^+^ cells) in infected vs saline-injected mice, regardless of *T. gondii* line (Fig. 5h–m). A lower inflammatory cell count in GRA16-HA-infected mice at 3 mpi was consistent with lower levels of *T. gondii* observed in the brain and lack of significant weight loss during the acute phase of infection, which suggests that they had a more limited infection that was cleared before reaching the brain (Fig. 5e–m and Extended Data Fig. 4). Collectively, these data emphasize that MeCP2 expression neither decreased the ability of the engineered *T. gondii* to reach and persist in the brain, nor did it alter the distribution of *T. gondii* in the body, the kinetics of its clearance from the periphery, its immunogenicity, or its specificity and levels of long-term persistence in the brain, all of which are well characterized for *T. gondii*^36–40^.

### 3D in vivo distribution of delivery in whole cleared brains

**Fig. 6:**
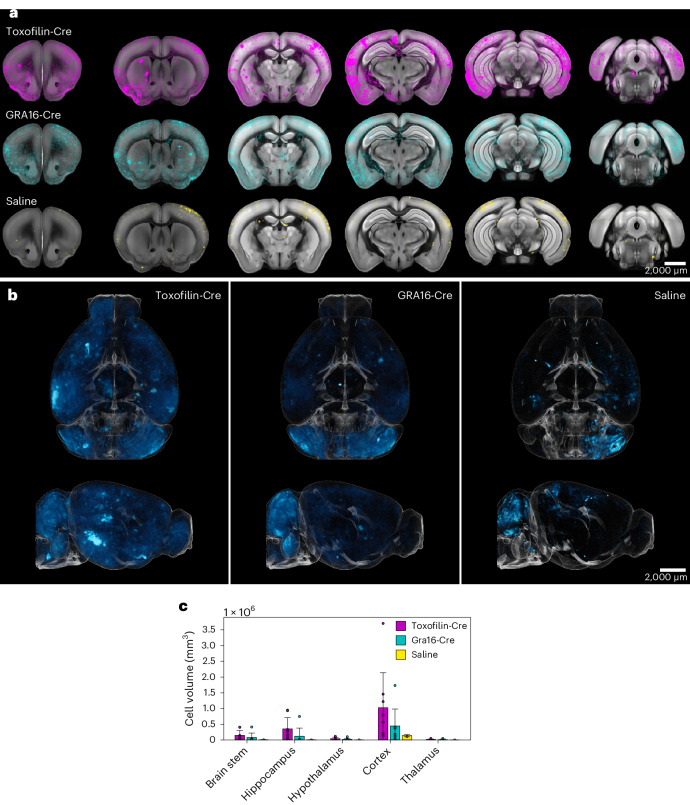
3D distribution of rhoptry- and dense granule-mediated protein delivery by *T. gondii* in cleared brains, following intraperitoneal administration in reporter mice. Ai6 reporter mice were injected intraperitoneally with toxofilin-Cre or GRA16-Cre Pru *T. gondii* or saline. At 21 dpi, brains were collected, processed, cleared using the CRYSTAL technique and imaged. **a**, Distribution of ZsGreen+ cells in different brain regions, averaged over all the samples in each group. Top: toxofilin-Cre (magenta); middle: GRA16-Cre (cyan); bottom: saline (yellow). **b**, 3D visualization of group-average distribution of ZsGreen+ cells within the whole brain. **c**, Total ZsGreen+ cell volume per brain region, obtained from the averaged data per group. Data represent mean ± s.d. Biologically independent repeats (mice) per group: *N* = 8 (toxofilin-Cre), 7 (GRA16-Cre) and 2 (saline). Scale bars, 2,000 μm.

**Extended Data Fig. 5:**
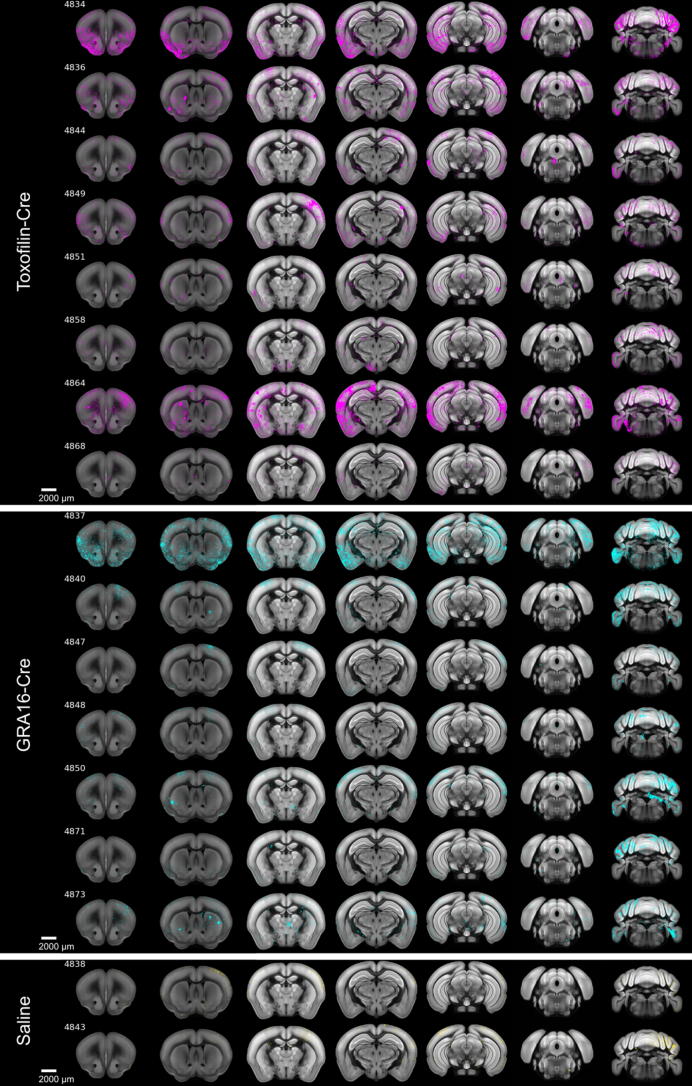
Per-mouse distribution of the fluorescent signal in mice treated with saline, GRA16-HA and GRA16-MeCP2 across 7 representative brain slices.

**Extended Data Fig. 6:**
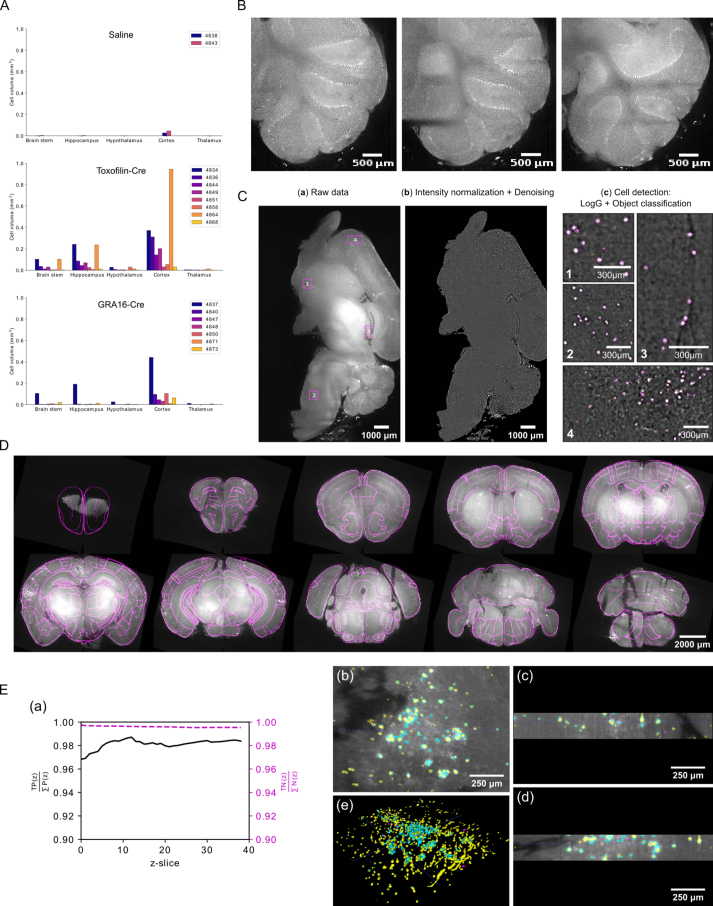
Per-mouse brain distribution quantification and extended methods for the CLARITY data processing, cell segmentation and alignment to the Allen brain atlas. (**A**) Distribution of rhoptry- and dense granule-mediated protein delivery by Pru T. gondii in different brain regions, cell volume per brain region, per animal in each group (mouse ID numbers are denoted in the legend). (**B**) Raw images of Purkinje neuron autofluorescence in the cerebellum of saline-injected control mice. The depicted slices are taken from sample 4843. The number of biologically independent samples in the saline group is N = 2. (**C**) Representative output from the 3D cell detection pipeline in whole-brains. All operations are three-dimensional, however for simplicity, we show here the output only at a slice of sample 4864. (a) Raw data. (b) Image restoration after intensity normalization and denoising. (c) Overlay of the perimeter of detected cells (magenta edge) and raw data. Numbers 1–4 correspond to the four boxes shown in (a). (**D**) Representative output of alignment to the Allen Brain Atlas. Coronal view of the aligned raw data (grayscale), onto the Atlas (magenta contour lines). Tiles correspond to slices of the coronal view at different depths of the sample. The output corresponds to sample 4864. (**E**) Validation of segmentation, with respect to manual annotations by domain experts. (a) Convergence of true positive (TP) and true negative (TN) pixels, with respect to the number of z-slices included in the computation. (b,c,d) overlay of segmentations and raw data, shown in slices along the (b) xy, (c) yz, and (d) xz plane. Color code: yellow: correctly detected cell core, cyan: non-detected boundary region around cell cores, magenta: false negatives. (e) 3D view of segmented data colored as described above. Images (b)-(e) are taken within the software ITK-SNAP (Yushkevich et al 2006).

To characterize the distribution of rhoptry- and dense granule-mediated protein delivery in the brain in more detail, we leveraged a Cre-lox system to fluorescently mark cells that receive rhoptry and dense granule proteins in vivo, on the basis of previously published lines with toxofilin-Cre and GRA16-Cre^16,19^. We inoculated Cre-reporter mice with a loxP-flanked STOP cassette controlling ZsGreen expression^41^. At 21 dpi, mice were euthanized, perfused with fixative and hydrogel monomer, and their brains were collected and processed by hydrogel polymerization and clearing following a variant of the active CLARITY (CRYSTAL) method^42^. Cleared whole brains were imaged on a mesoSPIM microscope, analysed using a custom pipeline for 3D cell segmentation (see Methods) and mapped onto the Allen Brain Atlas^43^ for statistical analysis of brain distribution (Extended Data Figs. 5 and 6). As expected, toxofilin-Cre was delivered to a substantially higher volume of neurons than GRA16-Cre, which requires establishment of an intracellular PV as a prerequisite for secretion (~2-fold difference) (Fig. 6a–c). Notably, we observed high variability in total cell volume delivered with the *T. gondii* proteins in each group (Extended Data Figs. 5 and 6), consistent with previous reports^36,39,40,44,45^. For both the rhoptry- and dense granule-secreted proteins, the highest level of protein delivery was observed in the cortex, followed by the hippocampus, brainstem, hypothalamus and thalamus (Fig. 6c, and Extended Data Figs. 5 and 6).

## Discussion

In this study, we show that *T. gondii* can be used to address many of the challenges associated with protein delivery for research and therapeutic applications. We demonstrate the use of *T. gondii* as a versatile delivery system in cultured fibroblasts, in vitro-differentiated neurons, primary neurons, human brain organoids and in vivo in mice, and characterize factors that affect delivery patterns under different conditions. Each of the two secretion systems tested harbours its own advantages. The rhoptry system secretes proteins by a ‘kiss-and-spit’ mechanism, discharging proteins directly to the host cytosol through a transient opening on the plasma membrane^46^. This enables injection of proteins to multiple cells by each individual *T. gondii* and does not necessitate cell invasion or persistence inside those cells^47^. In contrast, dense granule secretion requires *T. gondii* to be established within the host cell but can provide higher levels of protein secretion and longer-lasting delivery^48–51^. The benefits of each system may render them suitable for different kinds of protein delivery schemes.

Protein delivery from intracellular *T. gondii*, as is the case for dense granule secretion, involves additional challenges associated with the need to traverse the encapsulating PVM, for which the export mechanisms are not well understood but appear to be highly selective^22^. The three successfully exported heterologous fusion proteins in our study join the recently published GRA16-Cre line, which has been developed through parallel efforts in our lab^19^. A preliminary analysis (Extended Data Fig. 1) of the intrinsic disorder profile of the tested proteins aligns with existing models that relate variance in PVM transport with protein intrinsic disorder^51,52^, but the small sample size does not enable drawing of significant conclusions. We hope that these findings could support further improvement of protein delivery efficiency, or prioritization of different secretion mechanisms for different targets.

*T. gondii*’s ability to robustly deliver intracellular proteins to neurons emphasizes its potential as a research tool. Neurons are particularly difficult to target with existing methods, as they are less receptive to uptake of transfection reagents and to expression of exogenously delivered DNA. Their high sensitivity to changes in pH, osmolarity, physical stress and temperature makes many methods for intracellular delivery non-viable for neurons^53^. *T. gondii*-mediated intracellular delivery can be used as a tool to study protein activity in neurons, deliver genome-editing proteins to generate disease models, or deliver in vivo reporters^25,54,55^. *T. gondii*’s ability to deliver multiple proteins at once may be especially practical for this use. Simultaneous use of different carrier sequences, promoters and/or secretion systems enables orchestrating the delivery of proteins with different delivery kinetics, quantities, cell type targeting and localization. Importantly, the transgenic lines we developed were able to deliver surprisingly large full-length mammalian proteins into cells. When fused to the carrier proteins, the secreted proteins tested ranged 88–110 kDa in size. Although the sample size was small, there was no observed correlation between delivery efficiency and protein size, suggesting that protein size was not a limiting factor within this range. Furthermore, mechanistic understanding of the rhoptry and dense granule secretion systems does not point to such a limitation^22^. In contrast, the packaging limit of ~4.7 kb for adeno-associated viruses (AAV) and ~2.3 kb for self-complementary AAV (scAAV), which are currently the most commonly used viral vectors, severely constrains the range of proteins they can deliver^56,57^.

To illustrate the potential of this system, we developed it through the example use-case of neuronal protein replacement therapy, and in particular MeCP2. MeCP2 has been dubbed an ‘epigenetic reader’, but the precise ways in which it modulates gene expression and the epigenetic state of the cell are under debate^31,34,58–62^. Because of this, we performed a preliminary analysis of its functionality by combining several parallel assays, including (1) heterochromatin binding in mouse neurons in vitro and in vivo, (2) specific binding to methylated DNA in a biochemical pull-down assay and (3) transcriptional changes in human brain organoids. Notably, the transcriptional changes observed were relatively mild, and we believe this could be explained by (1) low number of infected cells due to a low multiplicity of infection, (2) short time between the delivery of the protein and sequencing, and (3) the complexity of the regulation by MeCP2, which affects the expression of many different genes both directly and indirectly and makes the changes it mediates highly context specific with high variance^31,34,58–62^. We discuss several ideas for addressing these limitations below. We provide additional evidence that *T. gondii*-delivered proteins retain their function with the Cre recombinase lines^16,19^. Toxofilin-Cre delivery activated Synaptophysin-TdTomato expression in primary neurons, and the toxofilin-Cre and GRA16-Cre lines activated ZsGreen in vivo. Using these lines, we also provide an in-depth characterization of the 3D distribution of protein delivery in cleared brains. Although each of these assays is preliminary and provides partial evidence by itself, we believe that together they provide strong support for the use of translational fusions to drive functional protein delivery with *T. gondii* in vitro and in vivo.

Many of the potential applications of this technology will depend on further increasing the amount of *T. gondii* or of the secreted protein in the brain. Potential improvements to the system may consist of (1) testing additional carrier proteins for rhoptry or dense granule delivery, (2) modifying their regulatory elements (for example, promoter and 3’UTR), (3) using different isolates of *T. gondii* with higher CNS targeting than the lab strains RH, Pru or ME49, (4) testing schemes for repeated dosing, (5) using proteins secreted by the bradyzoite stage^49,63^ or (6) increasing the inoculum of *T. gondii* administered, which will require developing attenuated strains.

**Extended Data Fig. 7:**
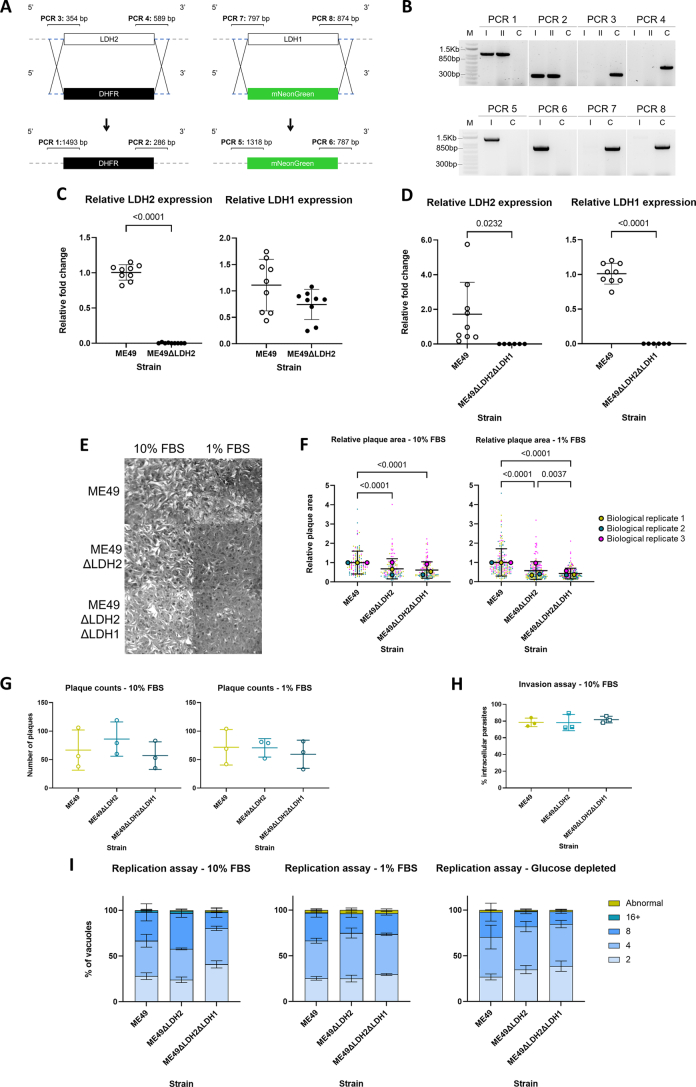
Mutant double-knockout of LDH1 and LDH2, which are reported to have inhibited growth in vivo, are viable and grow as well as WT T. gondii in vitro. (**A**) Scheme of the genetic constructs used in the sequential CRISPR-Cas9 mediated gene knock-out of LDH2 and LDH1 by Cas9-mediated insertional replacement with the selection markers DHFR (for LDH2) or mNeonGreen (for LDH2). We note the location of the different amplicons targeted by the diagnostic PCRs. (**B**) Gel electrophoresis results showing the amplicons from PCRs, spanning homology regions of insertion cassettes in ME49 T. gondii with the genetic knockouts ΔLDH2ΔLDH1 (I) and ΔLDH2 (II). The WT ME49 (**C**) parental strain is included as a control. M = MW marker (1 Kb Plus DNA Ladder). The numbers 1–8 above each gel correspond to the targeted PCR amplicons shown in (**A**).(**C, D**) Fold change in gene of interest mRNA expression relative to housekeeping gene Actin1 in ME49 and derived knockout mutants ME49ΔLDH2 (**C**) and ME49ΔLDH2ΔLDH1 (**D**). Comparative statistical analysis was performed using independent two tailed t-tests. Welch’s correction was used in the statistical analysis of ME49 vs. ME49ΔLDH2ΔLDH1. Error bars are Mean ± SD representative of three biological replicates. (**E**) Plaque assays comparing the growth of ME49 and derived knock-out mutants. 500 parasites were seeded into 6-well plates with DMEM supplemented with 10% or 1% FBS, and cultured for 7 days. (**F**) Relative plaque area for parasites grown in DMEM supplemented with 10% FBS (N = 130 plaques) or 1% FBS (N = 183 plaques). Relative plaque area analysis was done using Kruskal Wallis One-way ANOVA with Dun’s post hoc. Error bars are Mean ± SD representative of three biological replicates. (**G**) Number of plaques in a 307.88mm2 area for parasites grown in DMEM supplemented with either 10% FBS and 1% FBS. Plaque count analysis was done using One-way ANOVA with Tukey’s post hoc. Error bars are Mean ± SD, representative of three biological replicates. (**H**) Red-green invasion assays between ME49 and derived knock-out strains. Parasites were allowed to invade an HFF monolayer for 30 minutes prior to fixation and staining. Analysis was done via One-way ANOVA with Tukey’s post hoc. Error bars are Mean ± SD, representative of three biological replicates, consisting of two technical replicates each, N ≥ 100. (**I**) Parasite replication assays between ME49 and derived knock-out strains under different media culturing conditions. The coloring indicates the number of tachyzoites observed in each PV. Analysis done using One-way ANOVA with Tukey’s post hoc. Error bars are Mean ± SD, representative of three biological replicates, N = 150 vacuoles/condition. Source data

Exploring different ways of attenuating *T. gondii* will be one of the most important steps for developing any vectors based on *T. gondii*. Although natural infections in immunocompetent humans tend to be asymptomatic, *T. gondii* infections can still cause adverse effects in a variety of situations^12,37,64–66^, including evidence for neurotoxicity^44^. Analogous biological therapies that required strain attenuation include viral gene therapies^56^ as well as other live therapies utilizing natural pathogens such as listeria-based immunotherapies^67^, modified microbiome therapies^68^, helminth immunotherapies^69^, and live and heterologous vaccines^70–73^. Potential approaches may include disrupting virulence genes^74^, disrupting bradyzoite differentiation^75^, limiting in vivo replication or cell invasion which we are actively exploring (Extended Data Fig. 7)^76^, auxotrophy^77^, increased drug susceptibility^78^, increased sensitivity to the natural host immune response^79^ or inducible elimination in the form of synthetic circuits such as inducible ‘kill-switches’^80^. Overall, further studies that delineate and improve the safety and efficacy of *T. gondii*-based vectors will be necessary. We believe that ongoing advances in the development of genetic tools for *T. gondii*, improved characterization of the molecular mechanisms of infection, persistence and host-immunity, and the continuous interdisciplinary enrichment of approaches from other bioengineering fields will enable further exploration of the suitability of *T. gondii* as a vector and facilitate its development for varied applications.

## Methods

### *Toxoplasma gondii* culture and maintenance

Type I RH and type II Pru and ME49 strain *T. gondii* were grown in HFF in high-glucose Dulbecco’s modified Eagle’s medium (DMEM) supplemented with 4 mM l-glutamine, 10% fetal bovine serum (FBS) and 1% penicillin/streptomycin or 20 μg ml^−1^ gentamicin antibiotics (‘complete DMEM’) at 37 °C with 5% CO_2_. Cultures were monitored daily and *T. gondii* were passaged by transferring 1–3 drops (20–100 μl) of the supernatant of a lysed dish (containing extracellular parasites) into a fresh dish with confluent HFF cells. Type I RH and type II Pru strains were validated by PCR–restriction-fragment length polymorphism (primers described in Supplementary Table 1)^81^ or by passage into Cre Reporter cell lines to confirm Cre recombination as previously described^16^.

### Mice and administration of *T. gondii*

For all the experiments shown in Figs. 3, 5 and 6 and Extended Data Figs. 4a–i, 5 and 6, mice were housed in specific-pathogen-free University of Arizona Animal Care facilities in the following conditions: 14 h/10 h light/dark cycle, ambient temperature between 20–24 °C and humidity of 30–70%. Cre-reporter mice were originally purchased from the Jackson Laboratories: Ai6, 007906, B6.Cg-Gt(ROSA)26Sortm6(CAG-ZsGreen1)Hze/J and Ai34, 012570, B6;129S-Gt(ROSA)26Sortm34.1(CAG-Syp/tdTomato)Hze/J. For the infection experiment at 18 dpi, 36 mice aged 4–5 months (6 females and 6 males in each group) were inoculated intraperitoneally with 20,000 freshly syringe-lysed *T. gondii* tachyzoites diluted in 200 µl of UPS-grade phosphate buffered saline (PBS). At 18 dpi, mice were euthanized by CO_2_ asphyxiation and transcardially perfused with ice-cold PBS. For the infection experiment at 1 and 3 mpi, 33 mice aged 3–5 months (3–5 females and 5–7 males in each group) were inoculated as outlined above. At 1 or 3 mpi, mice were euthanized with a ketamine/xylazine cocktail and transcardially perfused with cold PBS. For the clearance experiment, 22 mice aged 4–6 months (5–6 females and 4–5 males per group with 2 saline controls) were inoculated intraperitoneally with 10,000 freshly syringe-lysed *T. gondii* tachyzoites diluted in 200 µl of UPS-grade PBS. At 21 dpi, mice were euthanized with a ketamine/xylazine cocktail and transcardially perfused with cold PBS followed by cold hydrogel as described in ‘Tissue preparation, clearing and refractive index matching’ below. All mouse studies and breeding were carried out in strict accordance with the Public Health Service Policy on Human Care and Use of Laboratory Animals. The protocol was approved by the University of Arizona Institutional Animal Care and Use Committee (#A-3248–01, protocol #12–391).

For the experiment in Extended Data Fig. 4j, the mice were housed at the CRO MD Biosciences in Ness Ziona, Israel in the following conditions: 12 h/12 h light/dark cycle, ambient temperature between 17–23 °C and humidity of 30–70%. Ten male C57BL/6J mice (Envigo RMS Israel) aged 4 weeks were injected through the lateral tail vein with 100 µl of UPS-grade PBS (*N* = 5) or 50,000 freshly syringe-lysed tachyzoites of ME49 GRA16-MeCP2 *T. gondii* diluted in PBS (*N* = 5). At 21 dpi, mice were euthanized by pentobarbital injection and their tissues were collected for DNA extraction. The protocol was approved by the Israeli Committee for Ethical Conduct in the Care and Use of Laboratory Animals.

### In vitro-differentiated LUHMES neurons

Culturing and differentiation of LUHMES cells were carried out according to refs. ^25,82^. In brief, LUHMES cells were maintained on flasks coated with fibronectin+poly-l-ornithine (1 μg ml^−1^ and 44 μg ml^−1^, respectively, in sterile water) at 37 °C with 5% CO_2_, with proliferation media consisting of advanced DMEM/F12 media supplemented with 1x N2 serum-free supplement, 2 mM l-glutamine and 40 ng ml^−1^ beta-FGF. Differentiation to neurons was induced by a differentiation medium that lacks beta-FGF and is supplemented with 1 μg ml^−1^ tetracycline, 2 ng ml^−1^ GDNF and 1 mM cell-permeable cAMP analogue (N6,2′-*O*-dibutyryladenosine 3′,5′-cyclic monophosphate sodium salt). LUHMES cells were split again the day after media change and then maintained for 6 more days. The cells were considered mature neurons from day 7 after initiation of differentiation, as confirmed by anti-NeuN staining. Wild-type vs MeCP2-KO LUHMES cells were validated by Sanger sequencing of the *MECP2* locus.

### N2A neuroblastoma cells

N2A (Neuro-2a, mouse neuroblastoma) cells were cultured in high-glucose DMEM supplemented with 10% FBS, 1% penicillin/streptomycin, 2 mM l-glutamine and 0.1 mM MEM non-essential amino acids solution at 37 °C with 5% CO_2_.

### Mouse primary neuronal cultures

The cortex and hippocampi of C57BL/6J MECP2^+/−^ (heterozygous) P1 pups were dissociated enzymatically using papain and mechanically using gentle pipetting. Cells were counted and seeded in a 24-well plate on poly-l-lysin-coated glass coverslips at a density of 100,000 cells per well, in neurobasal media (NBA, 2% B27 and 1% l-glutamine). After 5 days in culture, the neuronal cultures were inoculated with *T. gondii* tachyzoites.

Primary murine neurons from Synaptophysin-Tdtomato mice (Ai34(RCL-Syp/tdT)-D, Jackson Laboratories, 012570) were collected as previously described^19,83^. In brief, E17 brains were collected and made into a single-cell suspension with enzyme digestion and trituration. Approximately 100,000 cells were seeded onto poly-l-lysine-coated glass coverslips and grown for 8–9 days before infection with *T*. gondii.

### Human-induced pluripotent stem cells

Human-induced pluripotent stem cells (hiPSCs) have been previously validated in G.T.’s laboratory^84^. Before hiPSCs plating, cell culture dishes were coated for 10 min at 37 °C with a solution made of matrigel stock solution (Corning) diluted 1:40 in DMEM/F12 medium (Euroclone). Cells were cultured in TeSR/E8 medium (Stemcell Technology) supplemented with penicillin (100 U ml^−1^) and streptomycin (100 μg ml^−1^), with daily medium change at 37 °C, 5% CO_2_ and 3% O_2_ in standard incubators. hiPSCs were routinely split 1:6 to 1:10 using ReLeSR (Stemcell Technology) when confluency reached ~60–70%. When single-cell dissociation was needed, Accutase solution (Sigma-Aldrich) was used and 5 µM ROCK inhibitor (Sigma-Aldrich) was added in fresh medium to enhance cell survival. For cryopreservation, cells were dissociated when 60% confluent with Accutase and resuspended in TeSR/E8 medium supplemented with 10% dimethylsulfoxide and 5 µM ROCK inhibitor. All hiPSC lines employed were routinely checked for mycoplasma contamination and the correct cell line identity was confirmed through short tandem repeats profiling with the kit GenePrintR 10 system (Promega). Cell lines were profiled with Array-CGH to verify the absence of chromosomal rearrangements, using the Agilent kit SurePrint G3 Human CGH Microarray 8x60K.

### Cortical brain organoids

For the differentiation of cortical brain organoids, we used the protocol described in ref. ^85^, with minor modifications to improve its efficiency as shown previously by us^32,86^. Briefly, stem cells were grown in feeder-free conditions on plates coated with matrigel solution (Corning). For cortical brain organoids generation, hiPSC lines were cultured in 10 cm dishes up to 80% confluency. Then, the hiPSCs were dissociated into single cells with Accutase, centrifuged and resuspended in TeSR/E8 medium with 5 μM ROCK inhibitor Y-27632 (Tocris). The cell concentration was adjusted to 2 × 10^5^ live cells per ml after counting (viability cut-off for generation >90%). A volume of 100 μl per well of cell suspension was dispensed into U-bottom, PrimeSurface 96-well plates (SystemBio) before plates were centrifuged at 200 × *g* for 3 min to facilitate the aggregation of PSCs into embryoid bodies (EBs). The day of EBs generation is defined on the protocol timeline as differentiation day in vitro −2 (DIV −2). On DIV 0, media change was performed, replacing TeSR/E8 with differentiation medium 1 containing 80% DMEM/F12 (1:1), 20% Knockout serum (Gibco), 1 mM non-essential amino acids (Sigma), 2 mM GlutaMax (Gibco), 100 U ml^−1^ penicillin/100 μg ml^−1^ streptomycin (Sigma), 100 μM cell-culture-grade 2-mercaptoethanol and supplemented with 10 μM TGFbeta inhibitor SB431542 (MedChem Express) and 7 μM dorsomorphin (Sigma) for dual SMAD inhibition. Media change was performed once a day with 100 μl per well of medium 1 up to DIV 4. Starting DIV 5 onwards, media change was performed with differentiation medium 2 composed of Neurobasal medium without phenol red, containing B27 without vitamin A (Gibco 1:50), 100 U ml^−1^ penicillin/100 μgml^−1^ streptomycin (Sigma) and 100 μM cell-culture-grade 2-mercaptoethanol. From DIV 5 to DIV 24, differentiation medium 2 was supplemented with 20 ng ml^−1^ of basic fibroblast growth factor (bFGF) (Peprotech) and 20 ng ml^−1^ of epidermal growth factor (EGF) (Peprotech) to support the survival of neural progenitors. On DIV 12, cortical brain organoids were transferred into PrimeSurface 90 mm dishes and thereafter constantly shaken at 50 r.p.m. to facilitate diffusion of nutrients. From DIV 12 onwards, media changes were performed every other day. Since DIV 24 and up to DIV 43, EGF and bFGF in medium 2 were replaced with 20 ng ml^−1^ brain-derived neurotrophic factor (BDNF) (Peprotech) and 20 ng ml^−1^ of Neurotrophin-3 (Peprotech), whereas from DIV 43 onwards, medium 2 without growth factors was used. From DIV 12 onwards, the volume of media added at every media change was gradually increased to ensure organoids remained covered. On average, while on DIV 12, ~12–15 ml of medium were required for each 90 mm plate, from DIV 40 onwards, the required amount of medium stabilized around a maximum of 30 ml. For the experiments in this paper, cortical brain organoids were grown for 200 days for the immunofluorescence experiments and for 50 days for the single-cell transcriptomics. At 1 week before the infection experiments, organoids were moved to 6 multiwell plates filled with 5 ml of media per well.

### DNA transfection of *T. gondii*

Tachyzoites of *T. gondii* were collected from the supernatant of lysed cells or mechanically released by scraping and passing through a 23–27 gauge needle. Extracellular parasites were filtered using a 3-μm-pore filter (Whatman Nuclepore polycarbonate membranes, GE Healthcare, 110612), spun down for 5 min at 800 × *g* and resuspended in cytomix buffer (10 mM K_2_HPO_4_/KH_2_PO_4_, 25 mM HEPES, 2 mM EGTA pH 7.6, 120 mM KCl, 0.15 mM CaCl_2_, 5 mM MgCl_2_ with 5 mM KOH adjusted to pH 7.6) freshly supplemented with 3 mM ATP and 3 mM glutathione (GSH). Resuspended parasites (350–800 μl) were placed in an electroporation cuvette and supplemented with up to 50 μl of the transfected DNA (10–80 μg DNA). Electroporation was performed using two square-wave pulses, using either the BTX ECM 830 or Bio-Rad GenePulser Xcell electroporators. On the BTX electroporator, transfection was performed using the following settings: high voltage (HV) mode, voltage 1,700 V, pulse length 0.05 ms, 2 pulses, 200 ms interval, unipolar. On the Bio-Rad electroporator, transfection was performed using the following settings: programme 3 (square-wave protocol), voltage 1,700 V, pulse length 0.2 ms, 2 pulses, 5 s interval, 4 mm cuvette. The transfected parasites were transferred onto cells, fixed at 24 and 48 h after inoculation, immunofluorescently stained and microscopically assessed for transfection efficiency, protein expression and localization.

### Generation of *T. gondii* transgenic clonal lines

To generate stable clonal lines, *T. gondii* were transfected as described and genomic integration of the exogenous DNA was selected for by drug selection followed by clone isolation. For drug selection, transfected *T. gondii* were passaged in media containing pyrimethamine (1 μM for dihydrofolate reductase-thymidylate synthase (DHFR-TS) selection) or mycophenolic acid+Xanthine (25 μg ml^−1^ and 50 μg ml^−1^ respectively, for hypoxanthine-xanthine-guanine phosphoribosyl transferase (HXGPRT) positive selection). After 3 weeks of drug selection, the parasites were considered a ‘stable pool’ containing parasites that integrated the exogenous DNA construct into their genome. The stable pool was immunofluorescently stained to assess the levels of protein expression and localization in the stable pool. Clonal lines were isolated in 96-well plates with HFF by limiting dilutions or by FACS sorting, tested for genomic integration by PCR and verified by immunofluorescent staining. Protein localization was determined morphologically on the basis of comparison to the polarized light image and to co-staining with DAPI and anti-inner membrane complex 1 (anti-IMC1). Each fusion protein localization was validated over 2–5 independent transfections (Extended Data Table 2). At least 100 parasites were microscopically assessed by eye per transfection, and at least 20 *z*-stack images were acquired for each transfection, for transient and for stable (genomic) expression.

For the Pru GRA16-HA-MeCP2:TCre and parental TCre parasites, the procedure outlined above was followed except with a zeomycin selection marker. Post transfection, freshly egressed parasites were resuspended in DMEM supplemented with 50 μg ml^−1^ of Zeocin (InvivoGen, 11006-33-0) for 4 h and then added to HFF monolayers supplemented with 5 μg ml^−1^ Zeocin to select for integrants. This process was repeated three times before single-cell cloning by limiting dilution. Selected individual clones were confirmed to trigger Cre-mediated recombination using previously described methods^16,38^.

### Generation of *T. gondii LDH* mutants

Gene sequences were retrieved via ToxoDB (ToxoDB.org). Guide RNAs targeting sequences to be used for CRISPR-Cas9-mediated gene deletion were identified using CHOPCHOP (v.3)^87^, annealed and ligated into the pG474 CRISPR-Cas9 plasmid vector. Two guide sequences were designed for each gene: *LDH1* (TGME49_232350): aagttTTTCTCCTCTGCACAAGTGCg and aagttGAGCGCCATGACGTCGTCGAg; *LDH2* (TGME49_291040): aagttAATCTTTTTTCTTCTGCTAAg and aagttGGGTTGAACAAGTACGCAGGg. Insertion cassettes with homology overhang flanks were amplified from pDT7S4-G13M5 and pHL018-pBM014_SAG1-mNeonGreen_TUB1-dTomato (gift from Dr Clare Harding) template plasmids by PCR. *T. gondii* tachyzoites were transfected with plasmid vectors and DHFR insertion cassette by electroporation, and single *LDH2* knockout mutants were selected with 1 μM pyrimethamine. A clonal line was isolated and validated by diagnostic PCR. Secondary genetic deletion of *LDH1* was conducted utilizing the same CRISPR methodology but with an mNeonGreen insertion cassette. Fluorescent parasites were isolated by flow cytometry and a clonal line was validated by diagnostic PCR. RNA was extracted from parasite material cultured in routine conditions using an RNeasy mini kit (Qiagen). RNA concentration and quality was analysed using a NanoDrop 2000 spectrophotometer (Thermo Scientific) and concentrations normalized to <2 µg. RNA samples treated with DNase (Thermo Scientific) were converted to complementary DNA using a High-Capacity RNA-cDNA kit (Applied Biosystems, Thermo Scientific) according to manufacturer instructions, with an assumed completed reaction yielding an equivalent <2 µg cDNA. Reaction mixtures were made with 2X Power Sybr Green (Applied Biosystems, Thermo Scientific) and loaded onto MicroAmp Optical 96-well reaction plates before amplification via a 7500 Real Time PCR system (Applied Biosystems, Thermo Scientific), with data acquisition using the 7500 system software (Applied Biosystems, Thermo Scientific). Data were interpreted as relative expression using the double delta CT method.

Primer off-target binding and dimerization risk was mitigated using a combination of SnapGene (v.6, GSL Biotech) and OligoAnalyzer (Integrated DNA Technologies (IDT)). RT–qPCR primers were generated using the PrimerBLAST NCBI software with an optimal temperature of 57–63 °C, guanosine-cytosine content of 50–60% and amplicon spanning an exon–exon junction where applicable. Insertion cassette primers were designed to include a 40–50 nt region with homology to either 5’ or 3’ untranslated region of the gene of interest and ~20 nt region with homology to a template plasmid. RT–qPCR statistical analysis was conducted using GraphPad Prism v.7.0 for Windows (GraphPad Software).

### Generation of *T. gondii* with toxofilin-fused ZFNs and Cas9

Zinc Finger Nucleases act as heterodimers and therefore require the delivery of two separate proteins to the same cell (‘Left’ and ‘Right’). We tested three approaches for multiprotein delivery with *T. gondii*. The first approach consisted of co-transfecting two constructs to each parasite: one for the secretion of the Right-ZFN variant and one for the secretion of the Left-ZFN variant. The second approach consisted of developing two separate *T. gondii* strains, each delivering one of the two ZFNs and treating the cells with both parasite strains at the same time. The third approach consisted of *T. gondii* that express both ZFN variants fused in one long open reading frame, with an intervening T2A self-cleaving peptide that would separate them during protein translation (toxofilin-ZFN-L-T2A-toxofilin-ZFN-R). The constructs also included a 3xFlag epitope tag between toxofilin and the ZFN (for toxofilin-ZFN-R and toxofilin-ZFN-L), or both a 3xFlag and an HA tag (for toxofilin-ZFN-L-T2A-toxofilin-ZFN-R, one for each ZFN variant) (Fig. 1e). As a test case, we used ZFN-L and ZFN-R sequences that target EGFP, adapted from ref. ^88^.

In addition, we tested different approaches for generating *T. gondii* for delivery of the *S. pyogenes* Cas9 endonuclease (Fig. 1e). Targeting of Cas9 to specific genomic loci requires the successful assembly of a stable ribonucleoprotein (RNP) complex consisting of the Cas9 protein and single-guide RNA (sgRNA). Because it was unknown whether an RNP can correctly assemble in *T. gondii* in a way that allows it to be targeted and secreted by the rhoptries, we tested two different approaches. The first approach consisted of expressing both the Cas9 protein and the sgRNA in *T. gondii*. The gRNA expression cassette in this case was controlled by the *T. gondii* endogenous U6 promoter^89^. The second approach consisted of expressing only the Cas9 protein in *T. gondii* and introducing an additional plasmid driving the expression of the gRNA directly in the host cell by plasmid transfection of the host cells. The gRNA expression cassette in this case was controlled by the mammalian U6 promoter^90^. Targeting of the Cas9 protein to the rhoptries was achieved by fusion to toxofilin, with the addition of a 3xFlag tag between the two proteins. The gRNA used was adapted from ref. ^91^ and is designed to target the human *Cetn1* gene.

Overall, we tested 5 constructs in type I RH *T. gondii*: (1) toxofilin-ZFN-R only, (2) toxofilin-ZFN-L only, (3) co-transfection of toxofilin-ZFN-R and toxofilin-ZFN-L, (4) toxofilin-ZFN-L-T2A-toxofilin-ZFN-R and (5) toxofilin-Cas9 and toxofilin-Cas9 with the *T. gondii* gRNA cassette.

### Immunofluorescence staining of in vitro samples

Cells were grown on 13 mm glass coverslips in 24-well plates. At the respective timepoint, cells were fixed in 4% paraformaldehyde (PFA) for 20 min at room temperature (r.t.). They were permeabilized and blocked by incubation with 2–3% blocking solution (2% bovine serum albumin, 0.2% Triton X in PBS) for 20 min at r.t. Blocking solution was removed and coverslips were covered with primary antibodies in blocking buffer and incubated for 1 h at r.t. or overnight at 4 °C. Following 3 washes with PBS, they were covered with secondary antibodies in blocking buffer and incubated for 40–120 min at r.t., protected from light. Coverslips were washed three more times with PBS, dipped briefly in water, blotted on paper to remove excess water and mounted on slides with either Fluoromount-G mounting media containing DAPI (Southern Biotech, 0100-20) or Prolong Diamond (Life Techologies, P36970). Slides were allowed to dry overnight at r.t. before imaging. For long-term storage, slides were kept at 4 °C in the dark.

### Mouse tissue preparation and staining

At 18 dpi, 1 mpi and 3 mpi, mice were euthanized by CO_2_ asphyxiation or ketamine/xylene, followed by transcardial perfusion with ice-cold PBS. At 21 dpi, mice were euthanized by CO_2_ asphyxiation followed by transcardial perfusion with 20 ml cold PBS and then 20 ml cold hydrogel. Collected brain was processed for immunofluorescence as previously described^13,47^ or left in hydrogel at 4 °C overnight before being shipped to the Aguzzi lab. For immunofluorescence, in brief, the left half of the collected brain was drop fixed overnight (4 °C) in 4% PFA in phosphate buffer, followed by embedding in 30% sucrose. The brain was then sectioned into 40-μm-thick sagittal sections using a freezing sliding microtome (Microm HM 430). Sections were stored as free-floating sections in cryoprotective media (0.05 M sodium phosphate buffer containing 30% glycerol and 30% ethylene glycol) until stained and mounted on slides. Free-floating tissue sections were stained with the primary antibodies in 30% goat serum/0.3%TX/PBS, followed by incubation in appropriate secondary antibodies. For 1 and 3 mpi cohorts, samples of lung, heart, liver and spleen tissue were also collected and divided into three. One-third of the samples was fixed in 4% PFA and processed as above. One-third was submitted to the University Animal Care (UAC) Pathology Services Laboratory, University of Arizona for immersion-fixation in 10% buffered formalin, followed by paraffin embedding. Five-micron sections were stained with haematoxylin and eosin (H&E). The last third of the samples was flash frozen and stored at −80 °C until used for isolating DNA. To quantify the total levels of *T. gondii* in the different organs, we performed qPCR on DNA extracted from the tissues, using primers targeting a parasite-specific gene (*B1*)^92^ and used the mouse *GAPDH* as a normalization control.

### Antibodies and dyes

Antibodies and/or reagents and the respective concentrations they were used in, are as follows: for in vitro immunofluorescence staining: anti-HA (Sigma-Aldrich, ROAHAHA/Roche clone 3F10, 1:1,000), anti-IMC1 (gift from Prof. Dominique Soldati-Favre, 1:2,000), anti-MeCP2 (Cell Signaling Technology, 3456, 1:200), anti-NeuN (Abcam, ab104224, 1:500), anti-ROP2/4 (gift from Prof. Dominique Soldati-Favre, 1:500), Alexa Fluor goat anti-rat 488 and 594 (Invitrogen, A-11006, A-11007, 1:1,000), Alexa Fluor goat anti-rabbit 488 and 594 (Invitrogen, A-11008, A-11012, 1:1,000), Alexa Fluor anti-mouse 488 and 594 (Invitrogen, A-11001, A-11005, 1:1,000). For tissue immunofluorescence staining: fluorescein-labelled Dolichos biflorus agglutinin (DBA) (Vector Laboratories, FL-1031, 1:500), 4’,6-diamidino-2-phenylindole, Dilactate (DAPI) (Life Technologies, D3571, 1:500), rabbit anti-HA-Tag (Cell Signaling Technology, C29F4, 1:1,600), biotin conjugated anti-NeuN (Millipore, MAB377B, 1:500). For tissue (3,3’-diaminobenzidine (DAB)) staining: anti-Iba-1 (019–19741, Wako, 1:3,000), anti-mouse CD3ε 500A2 (550277, BD Pharmingen, 1:300), biotinylated goat anti-rabbit (BA-1000, Vector Laboratories, 1:500) and biotinylated goat anti-hamster (BA-9100, Vector Laboratories, 1:500). Sections were then incubated in avidin-biotin complex (ABC) staining reagent (32020, ThermoFisher) for 1 h, followed by DAB (SK-4100, Vector Laboratories) detection of biotinylated antibodies.

### Microscopy data acquisition and analysis

Unless specified otherwise, all slides of the in vitro samples were imaged using the DeltaVision Core microscope (AppliedPrecision) using a ×100 objective. Images were handled using the Fiji distribution of ImageJ, imported using the OME bio-formats plugin and deconvolved using the Diffraction PSF 3D and Iterative Deconvolution 3D plugins^93^. All adjustments of brightness and contrast were linear and applied to the entire image equally. Background fluorescence was determined by sampling an ‘empty’ area of the image, and maximum display threshold for the image was set to allow optimal visualization of the cell structures and protein localization. All raw images, imaging metadata and ImageJ macros detailing the processing protocol are available in GitHub^94^.

For the brain tissue cyst quantification, the numbers of cysts were enumerated for 7 or 9 sections per mouse using a standard epifluorescent microscope (EVOS microscope). Only objects that stained for DBA and showed DAPI staining consistent with parasite nuclei were quantified as cysts.

For the immunofluorescence assays on brain sections, an inverted Leica SP5-II resonant scanner confocal microscope (University of Arizona, Cancer Center Microscopy Core) with standard Leica LAS AF software (v.2.7.3.9723) was used. All images shown in a given figure and with a given colour were obtained using identical parameters. For each field of view (FOV), 10 images per *z*-stack were taken with 2.5–3 μm between images. Images were rendered in ImageJ and analysed for the co-localization of NeuN and HA.

To quantify the number of CD3ε^+^ cells, for each brain section, 12 FOVs were taken in a stereotyped manner across the cortex (36 images in total per mouse). Images were taken with an Echo Revolve microscope (R4 from Echo Laboratories) (courtesy of Dr Anne Wertheimer from the University of Arizona in the BIO5 Institute, with funding from TRIF (Technology and Research Initiative Fund) and the Equipment Enhancement Fund). CD3^+^ cells per FOV were quantified by manually counting cells using ImageJ software. For Iba1^+^ cells, the same procedure was followed, except that at 1 mpi, 4 FOVs per brain section per mouse were captured and analysed. The 4 FOVs summed to the equivalent area of the 12 FOVs. For both CD3 and Iba1, the analyses were performed on 3 brain sections per mouse. The resulting numbers were averaged afterwards to obtain the average number of Iba1^+^ or CD3^+^ cells per brain section per mouse. Investigators quantifying CD3^+^ and Iba1^+^ cells were blinded to the infection status of the mouse until after the counts were completed.

The H&E-stained sections were analysed by light microscopy. To sample each of the tissue sections in a stereotyped way, 8 FOVs of the heart/liver/lungs per mouse were imaged and analysed. The degree of inflammation was assessed as follows: 0, no inflammation; 1, moderate inflammation; and 2, high inflammation. Then, for each tissue type, scores of the individual FOVs per mouse were averaged to yield an average inflammation score per mouse. Investigators assigning inflammation scores for the tissue samples were blinded to infection status of the mouse until after data were collected.

### Quantitative real time PCR of parasite DNA in vivo

To quantify parasite burden, we isolated genomic DNA from the rostral quarter of the frozen brain using the DNeasy Blood and Tissue kit (69504, Qiagen) and following manufacturer protocol. Next, amplification of the *Toxoplasma*-specific, highly conserved 35-repeat *B1* gene was performed using SYBR Green fluorescence detection with the Eppendorf Mastercycler ep realplex 2.2 system. *GAPDH* was used as a house-keeping gene, with primers and results calculated as previously described^92,95^.

### Measuring host nuclear fluorescence intensity in cells

Cells infected with *T. gondii* expressing the respective construct were fixed at the designated timepoints (24 h for HFF, 12–24 h for LUHMES) and immunofluorescently stained as described. Infected HFF were stained with anti-HA and anti-IMC1 (for staining the heterologous protein and *T. gondii* cell outline), and infected LUHMES neurons were stained with anti-HA and anti-NeuN (for staining the heterologous protein and verifying neuron differentiation) or with anti-HA and anti-MeCP2 (for quantifying nuclear MeCP2 in infected neurons), and imaged on the microscope. Mean nuclear intensity of the fluorescent signal of interest was measured using imageJ’s ‘Measure’ function. HA nuclear fluorescence was normalized by subtraction of the average image background sampled from empty regions in the image. MeCP2 nuclear fluorescence was normalized by subtraction of the nuclear fluorescence recorded from uninfected MECP2-KO neurons.

### Intrinsic disorder scoring and analysis

The intrinsic disorder profile for the full translated open reading frames of each GRA16 fusion protein was calculated using https://iupred2a.elte.hu/, with the following parameters: prediction type IUPred2 long disorder (default), context-dependent prediction: ANCHOR2 (ref. ^96^). A custom Python code was used to calculate the averaged intrinsic disorder score of the fused therapeutic protein, calculate the correlation between the intrinsic disorder score and nuclear localization of each fusion protein, and generate plots. The averaged intrinsic disorder score was calculated as the average of the ANCHOR2 score and the IUPred2 score along the span of the fused heterologous protein (excluding GRA16-HA), minus the 0.5 threshold for disorder. Protein disorder data and code are available in GitHub^94^.

### Automated high-content imaging of protein secretion in HFFs

HFF cells were seeded in five 384-microwell plates corresponding to five fixation timepoints (8, 16, 24, 32, 40 h) at a dilution of 3,500 cells per well in 50 μl complete DMEM, and given 2 days to reach confluency (4,500 cells per well). Tachyzoites of the lines GRA16-HA, GRA16-MeCP2 and GRA16-TFEB were collected and filtered. Wells were infected at MOIs of 3, 1 or 0.33 (13,500, 4,500 or 1,500 parasites in 20 μl per well, respectively), or received media only (MOI 0). At each timepoint, the respective plate was washed with PBS and fixed with 4% PFA. Each condition (parasite line+MOI+timepoint) was repeated over 24–40 wells. Immunostaining was performed using a Beckman Coulter Biomek FXp liquid handling robot with a Thermo Multidrop reagent dispenser and with a MANTIS liquid handler. Cells were first permeabilized and blocked with 2% blocking solution for 20 min at r.t. Cells were then incubated with primary antibodies (anti-HA and anti-IMC1) for 1 h at r.t., washed three times with PBS, incubated with secondary antibodies and Hoechst 33342 dye (diluted 1:50,000) for 40 min at r.t. and washed. Plates were imaged on the GE IN-Cell 2000 platform, with 5 FOVs acquired from each well (average of 360 cells per FOV). To validate the high-content imaging assay, we performed a parallel manual analysis on a limited sample of the data and compared the results to those from the automated pipeline. The manual analysis demonstrated similar infection and protein delivery values, with slightly higher nuclear delivery in infected cells (84–94%, compared with 73–86%), probably due to more sensitive identification of infected cells achieved by manual labelling (Fig. 3k,l).

### Manual imaging of infection and secretion in LUHMES

LUHMES cells were differentiated to neurons in 24-well plates with glass coverslips using the protocol described above. On day 6 of differentiation, tachyzoites of the line GRA16-HA were administered at MOIs of 0.5, 1 or 2. At each timepoint (6, 12, 24 and 32 h post inoculation), the respective wells were washed with PBS, fixed and immunofluorescently stained with anti-HA and anti-IMC1, or anti-HA and anti-NeuN manually as described. Each condition (MOI+timepoint) was repeated over 3 coverslips. Random regions of interest (10–60; average of 80 neurons per image) were imaged for each coverslip on the BX63 Olympus microscope using a ×40 objective.

### Analysis of infection and secretion in HFF and LUHMES

Image analysis was performed using the open-source CellProfiler software^97^ and resulting data tables were analysed using a custom Python code. Full protocol used for the image analysis, including the parameters chosen for identification of host cells and intracellular *T. gondii*, raw imaging data, image analysis output data tables and downstream analysis code are available in GitHub^94^. Briefly, host cell nuclei were identified using the DAPI channel and *T. gondii* were identified using the 594 nm channel (corresponding to anti-IMC1 staining). Morphological features were extracted for each identified host cell nucleus and parasitophorous vacuoles. Each *T. gondii* vacuole was associated with a host cell nucleus on the basis of proximity. Fluorescence intensity on the 488 nm channel, corresponding to the anti-HA staining, was used to quantify the levels of GRA16 fusion-protein localization in the parasitophorous vacuoles and in the host cell nuclei. Importantly, timepoints above 24 h post inoculation were removed from the dataset as we found that the parasite vacuoles were too large for efficient segmentation and host cell association by the used image analysis tool. Downstream analysis involved labelling and organization of the data, removal of images in which parasite identification failed (infected wells with no identified vacuoles or more than 400 identified vacuoles), removal of wells with extreme outlier fluorescence intensity (>5 s.d. from mean of condition), removal of wells with outlier numbers of identified parasitophorous vacuoles (>3 s.d. from mean of condition), normalization by subtraction of background fluorescence, labelling of cells infected with a single parasite vacuole and labelling of nuclei positive for the tagged protein (threshold set as above 99% of uninfected cells). Descriptive statistics were calculated for each well (*N* = number of cells in each well) and for each condition on the basis of the means of all wells treated in the same condition (*N* = number of wells in each condition) and plotted.

### Statistics and reproducibility

For every new toxofilin or GRA16 fusion construct generated in this study and presented in Figs. 1 and 2, localization was assessed over at least 2 and up to 5 independent transfections. The number of independent transfections (*N*) performed for each construct is listed in Extended Data Table 2 and also provided here: toxofilin-HA-ASPA (*N* = 5), toxofilin-HA-ASPAopt (*N* = 3), toxofilin-HA-MECP2 (*N* = 4), toxofilin-HA-MECP2opt (*N* = 4), toxofilin-HA-GALC (*N* = 4), toxofilin-HA-GALCopt (*N* = 2), toxofilin-HA-GALC-TAT (*N* = 4), toxofilin-HA-SMN1 (*N* = 4), toxofilin-HA-GDNF (*N* = 2), toxofilin-HA-PARK2 (*N* = 2), toxofilin-HA-TFEBopt (*N* = 3), toxofilin-ZFN-R (*N* = 2), toxofilin-ZFN-L (*N* = 2), toxofilin-ZFN-L-T2A-toxofilin-ZFN-R (*N* = 2), toxofilin-Cas9 (*N* = 2), toxofilin-Cas9_gRNA (*N* = 2), GRA16-HA-ASPA (*N* = 3), GRA16-HA-ASPAopt (*N* = 3), GRA16-HA-GALC (*N* = 3), GRA16-HA-GALCopt (*N* = 2), GRA16-HA-GALC-TAT (*N* = 4), GRA16-HA-MECP2opt (*N* = 4), GRA16-HA-SMN1 (*N* = 2) and GRA16-HA-TFEBopt (*N* = 4).

For the manual quantification of protein delivery used to validate the automated high-content imaging pipeline in HFFs, the number of repeats shown in Fig. 3l and used for the statistical analysis corresponds to the number of cells quantified for each condition (*T. gondii* line and time). The number of repeats for each condition (*N*) are as follows: 0 h post infection (hpi): *N* = 246; GRA16-HA: 8 hpi (*N* = 123), 16 hpi (*N* = 126), 24 hpi (*N* = 151); GRA16-MECP2: 8 hpi (*N* = 84), 16 hpi (*N* = 147), 24 hpi (*N* = 133); GRA16-TFEB: 8 hpi (*N* = 110), 16 hpi (*N* = 167), 24 hpi source d (*N* = 104). The full raw data are provided in the Source data file.

For the automated high-content imaging pipeline in neurons, the number of repeats shown in Fig. 3n and used for the statistical analysis corresponds to the number of images quantified for each condition (MOI and time). The number of repeats for each condition (*N*) are as follows: 0 hpi (*N* = 60); MOI 0.5: 6 hpi (*N* = 60), 12 hpi (*N* = 40), 24 hpi (*N* = 40); MOI 1: 6 hpi (*N* = 39), 12 hpi (*N* = 40), 24 hpi (*N* = 10); MOI 2: 6 hpi (*N* = 21), 12 hpi (*N* = 40), 24 hpi (*N* = 10). The full raw data are provided in the Source data file.

Statistical analysis for the differences in nuclear fluorescence intensity was performed using GraphPad Prism v.7.0 for Windows. To determine the significance of nuclear localization for each GRA16-fused protein, we performed one-way analysis of variance (ANOVA) for the effect of the expressed construct, with multiple comparisons to the ‘no construct’ control. To determine the significance of nuclear localization for each truncated variant of GRA16 fused to HA or to HA-MeCP2, we performed two-way ANOVA for the effect of the GRA16 variant (rows) and of the fused sequence (HA or HA-MECP2, columns), with multiple comparisons to the ‘no construct’ control. To determine the significance of nuclear levels of MeCP2 and GRA16-MeCP2 in LUHMES neurons, we performed two-way ANOVA for the effect of the time after inoculation (rows) and the condition (columns), with multiple comparisons to the ‘uninfected *MECP2*-KO’ control. Multiple comparisons were performed using the following parameters: within each row, compare the means of each group to control, report multiplicity adjusted *P* value for each comparison (Dunnett test), one family per row.

For statistical analysis of the data generated from the high-content imaging experiment, we used the SciPy and StatModels packages in Python. To determine the significance of the differences in infection and protein delivery between parasite lines, we performed three-way ANOVA for the effect of parasite line, MOI and time on the predicted parameter (infection rate, average number of parasite vacuoles per cell, percentage of infected cell nuclei positive for the tagged protein or normalized mean nuclear intensity of the tagged protein fluorescence). The two *P* values reported in the text for each parameter show the effect of GRA16-MeCP2 or GRA16-TFEB (categorical variables) compared to GRA16-HA. To determine the doubling rate, we performed log_2_ transformation on the ‘vacuole area’ values and fit a linear regression model over time. The doubling rate was calculated as one divided by the slope of the regression line. To determine the significance of the difference in doubling rate between parasite lines, we performed three-way ANOVA for the effect of parasite line, MOI and time on the log_2_-transformed vacuole area values. To compare the effect of the parasite line on the slope (change in area over time), the reported *P* values show the effect of the interactions between time and GRA16-MeCP2 or GRA16-TFEB (categorical variables) compared to GRA16-HA. Source data used for statistical analysis and plotting are available in the Source data file. Code used for statistical analysis of the HFF and LUHMES kinetics dataset is available in GitHub^94^.

### Preparation of protein lysates for DNA pull-down assay

HFF cells inoculated with *T. gondii* were washed with PBS, dissociated with Accutase solution and washed again with ice-cold PBS. Pellets were lysed with MPER lysis buffer (78501, ThermoFisher) with protease inhibitor cocktail (PIC, 100X, P830, Merck) for 5 min on ice, followed by 30 min on a rotator (Elmi Intelli-Mixer RM-2, Miliot Science) at 4 °C. Samples were centrifuged (16 kg for 15 min at 4 °C) and the supernatant was concentrated by centrifuging again in 0.5 ml Amicon 50 kDa concentrators (MMUFC505024, Millipore) at 4,000 *g* for 5 min. The 50 kDa concentrator was used to reduce competitive inhibition of binding from the endogenous human MeCP2 over GRA16-MeCP2. Of the pre- and post-concentration samples, 10 µl were used to measure protein concentration using a bicinchoninic acid (BCA) assay (23225, Pierce).

### Methylated and biotin-labelled DNA probes synthesis

Methylated and non-methylated DNA probes were generated by amplifying a DNA template with high GC content using biotin-labelled primers and methylated deoxynucleotides (dNTP) or non-methylated dNTP as a control. The template sequence used was: ACGTATATACGATTTACGTTATACGATTACGATATACGATTTACGTTAATACGTTTACGATTATTACGAATTTACGTTTTTACGAATATACGAAATACGTTTAATACGTAATTACGTATATTACGTATATACGATTTACGAATTACG. For methylated probes, we used methylated nucleotides (D1030, Zymo Research), and for non-methylated probes we used the dNTPs provided with the polymerase kit (Phusion, 0530, NEB). We used biotin-labelled primers (IDT), mCG F-biotin primer: 5´-biotin-cgtatatacgatttacgttatacga and mCG R-biotin primer: 5´-biotin-cgtaattcgtaaatcgtatatacgt. The probes were generated using 20 ×50 µl reactions and the expected amplicon size (147 bp) was confirmed by gel electrophoresis. The product was purified using the Clean and Concentrate Zymogen kit (D4004, Zymo Research) and eluted in 50 µl.

### Magnetic pull-down protocol using biotinylated DNA probes

The magnetic bead pull-down protocol was adapted from ref. ^98^, with the following modifications: (1) the nuclear lysate was added in the presence of LightShift Poly (dIdC) (20148E, Thermo Scientific), which was used here as a competitor for non-specific DNA binding proteins; and (2) incubation time of the nuclear lysate with the DNA probes was 30 min instead of 15 min. The magnetic beads were resuspended by vortexing and 8 µl was transferred to low protein-binding assay tubes. Beads were resuspended in 1 ml of PBS 0.1% Triton X-100 to each tube, then placed on the magnet and the liquid was removed. The beads were resuspended with 200 ng of the methylated/non-methylated probes (with 5’-biotinylated ends) in a total volume of 300 µl of PBS and incubated overnight at 4 °C with mixing to immobilize the probes to the beads. The beads were then washed three times with the wash solution, twice with PBS 1% Triton X-100 and three more times with a wash buffer. Then, the bound beads were resuspended with 40, 60 and 80 µg of the protein lysate + 250 ng poly-dIdC (0.25 µl) in a total volume of 300 µl of precipitation buffer. The assay tubes (lysates+probes) were incubated with mixing for 30 min at 4 °C (instead of 15 min in the referenced protocol). From each assay’s lysate, 10% was transferred to the input control tubes and then kept on ice. The beads were then washed five times with a wash buffer and once with PBS. After removal of the last wash, the beads or input samples were resuspended in sample buffer mix (5 µl of 4x LPS sample buffer (NP0007, ThermoFisher), 1.6 µl 1 M dithiothreitol in water to a volume of 20 µl), spun down and incubated on a heat block at 95 °C for 10 min and then kept on ice. The entire samples (input lysate or supernatant of the beads after heat elution) were loaded and run on a protein gel (NuPAGE 3–8%, Tris-acetate, EA0375PK2, Invitrogen), alongside a protein marker (9597580SM2700, Bio-Lab). The protein was transferred to nitrocellulose membrane using Bio-Rad’s transfer kit (1704158, Bio-Rad) and machine (1704150, Bio-Rad) using the mixed kDa programme which is 7 min long. The membrane was blocked in tris-buffered saline (TBST) 5% BSA for 1 h at r.t. on a shaker. The membrane was incubated in 1st Ab solution overnight, with shaking at 4 °C (1:1,000 in TBST 5% BSA, rabbit anti-MeCP2 (MeCP2 (D4F3) XP rabbit mAb, 3456, Cell Signaling Technology) in a total volume of 15 ml). The next day, the membrane was washed three times with TBST and then incubated for 1 h at r.t. with the 2nd Ab solution (111-035-144, Jackson Labs, 1:5,000 in TBST 5% BSA, in 10 ml). The membranes were incubated with an HRP substrate (WBLUF0100, Millipore) for 2 min in the dark and then imaged using a chemiluminescent western blot imaging device (AZI300, Azure).

### Infection of cortical brain organoids with *T. gondii*

All *T. gondii* strains were thawed and expanded on HFF in 10 cm dishes, according to previously published protocol^99^. To test the ability of *T. gondii* to infect and induce lysis of the organoids, when *T. gondii* strains had lysed the HFF monolayer and had been released extracellularly, cells were counted and 1 ml of each strain at a concentration of 1.5 × 10^6^ cells per ml was added to different wells of the organoids growing in 6 multiwell plates. After 5 days, infected organoids started to disintegrate and release *T. gondii* extracellularly. For this reason, the experiment was repeated following the same conditions for all the 3 strains, but organoids were collected 3 days after infection for immunofluorescence staining. For single-cell transcriptomics profiling, 1 ml of each *T. gondii* strain at a concentration of 1.5 × 10^7^ cells per ml was added to different wells and organoids collected after 24 h.

### Immunofluorescence of cortical brain organoids

Cortical brain organoids representing the 4 infection conditions: control unexposed organoids (CNT), organoids infected with the parental *T. gondii* (Toxo-dx), organoids infected with GRA16-HA *T. gondii* (Toxo-HA) and organoids infected with GRA16-Mecp2 *T. gondii* (Toxo-Mecp2) were collected on day 200 of organoids differentiation, 3 days after *T. gondii* infection, washed with PBS and fixed overnight at 4 °C in 4% PFA in PBS solution (SantaCruz). Fixed organoids were washed twice with PBS and mounted on OCT cryopreservation medium on dry ice. Cryoblocks were preserved at −80 °C until the moment of sectioning. Cryosections with 10 μm thickness were prepared using a Leica CM 1900 instrument. Sections were incubated with 10 mM sodium citrate buffer (Normapur) for 45 min at 95 °C with 0.05% Tween-20 for simultaneous antigen retrieval and permeabilization, then left to cool for at least 2 h at r.t. To immunolabel the markers of interest, a blocking solution made of 5% donkey serum (ImmunoResearch) in PBS was applied for 30 min to the slides, while primary antibodies diluted in blocking solution were subsequently added, followed by overnight incubation at 4 °C. Secondary antibodies and DAPI were diluted in PBS and applied to the sections for 2 h and 5 min, respectively. After each incubation step, 3 × 5 min washing steps with PBS buffer were performed. After a final rinse in deionized water, slides were air dried and mounted using Mowiol mounting medium. The following primary antibodies and dilutions were used: anti-toxoplasma, 1:200 (Abcam, Ab138698); anti-SOX2, 1:250 (R&D system, AF2018); anti-MAP2B, 1:250 (BD Biosciences, 610460); anti-TUBB3, 1:400 (Biolegend, 801202); anti-haemagglutinin (HA), 1:200 (Roche, 11867423001); anti-MeCP2, 1:200 (Cell Signaling Technology, 3456T); anti-NeuN, 1:200 (Millipore, MAB377). Images were acquired with a Leica DMI 6000B microscope (×100 objective), and processed and analysed using ImageJ (v.1.49) to adjust contrast for optimal RGB rendering.

### Single-cell RNA-seq

Single-cell transcriptomic data are publicly available and accessible through accession number PRJNA934842. All bioinformatic analyses and code to reproduce the figures are available via GitHub^100^ to ensure complete reproducibility and help the reader to consult, understand and re-use our data and analytical pipelines. Cortical organoids representing 3 infection conditions: control unexposed organoids (CNT), organoids infected with GRA16-HA *T. gondii* (Toxo-HA) and organoids infected with GRA16-Mecp2 *T. gondii* (Toxo-Mecp2) were collected on day 50 of organoids differentiation, 24 h after infection. For each condition, organoids were washed with PBS and dissociated with papain (STEMCELL Technologies) following manufacturer protocol (https://www.stemcell.com/how-to-dissociate-3d-neural-organoids-into-single-cell-suspension.html). For each of the 3 conditions, 3 replicates (single-cell suspensions dissociated from 3 different organoids) were independently labelled with oligo-barcoded lipids, following 10X CellPlex protocol (CG00391 Rev A protocol). Droplet-based single-cell partitioning and single-cell RNA-seq libraries were generated using the Chromium Next GEM Single Cell 3ʹ reagent kits (v.3.1 Chemistry) (10X Genomics) following manufacturer instructions. For each of the 3 reactions, concentration and volume of the single-cell suspension were loaded according to 10X Genomics indications to target 15,000 cells. The libraries were loaded on an Illumina NovaSeq 6000 system, with sequencing settings recommended by 10X Genomics (440 pM loading concentration) and a target coverage of 50,000 reads per cell. Single-cell transcriptome analysis: demultiplexing of the raw data was performed using CellRanger software v.7.0.0 from 10X Genomics (the command mkfastq that wraps Illumina’s bcl2fastq). The reads obtained from the demultiplexing were used as the input for ‘cellranger multi’ (CellRanger software v.6.1.2), which aligns the reads to reference genomes using STAR and collapses them to unique molecular identifier (UMI) counts. The reference genomes of the alignment included: the human genome GRCh38 distributed with cellranger (release 2020-A), the *T. gondii* ME49 genome (release 52) from ToxoDB (ToxoDB.org) and the sequences of the constructs used to engineer the *T. gondii* strains (GRA16-HA and GRA16-Mecp2). For demultiplexing of CellPlex barcodes, we retrieved the raw CMOs count matrix computed by CellRanger software using the multi pipeline and we kept cells that were not filtered out according to the barcode rank plot. Sample identities were untangled using the deMULTIplex pipeline (https://github.com/chris-mcginnis-ucsf/MULTI-seq) with automatic detection of the quantiles for threshold estimation at each iteration of assignment. After classification, cells identified as doublets or negatives in the final result were discarded. Standardized pipelines for filtering, normalization, dimensionality reduction, clustering and annotation of neurodevelopmental cell populations were used^101^ to analyse the resulting 57,818 cells (18,124 from the 3 replicates of CNT, 21,843 from the 3 replicates of Toxo-HA infected organoids and 17,851 from the 3 replicates of Toxo-Mecp2 infected organoids). To discard low-quality cells, barcodes were filtered according to the following criteria: (1) identified as ‘negative’ or ‘doublet’ by deMULTIplex; (2) number of detected genes lower than 1,000 or higher than 8,000, number of UMI lower than 2,000 or higher than 30,000; (3) % of mitochondrial counts higher than 15%, % ribosomal protein counts higher than 35%, % of human gene counts lower than 50%. Genes expressed in less than 200 cells were also filtered out. A total of 25,321 cells and 16,774 genes were selected for downstream analyses. Detection of highly variable genes was performed using Triku^102^. Aligning the single-cell transcriptomes to both human and *T. gondii* genomes, as well as the synthetic constructs used to generate the transgenic *T. gondii*, we could identify bona fide infected cells on the basis of three criteria: (1) cells with a significant amount of *T. gondii* transcripts, (2) cells with transcripts aligning to the synthetic constructs used to engineer the *T. gondii* and (3) cells that clustered together with cells that express high levels of *T. gondii* genes in a uniform manifold approximation and projection (UMAP) based on human and *T. gondii* genes quantification. All cells labelled as bona fide infected cells belonged to the experimental batches of the infected organoids, supporting the accuracy of this categorization (Extended Data Fig. 3).

The main analysis was performed considering only human genes for downstream calculations. We analysed the single cells following dimensionality reduction (principal component analysis, UMAP, diffusion maps) including only human gene quantification to avoid the noise in the transcriptomic analysis given by *T. gondii* transcripts that we previously used as markers for infection (Extended Data Fig. 3 and Table 3). UMAP dimensionality reduction as implemented in Scanpy^103^ was applied. Clusters were identified by applying the Leiden algorithm from Scanpy, which is a community detection algorithm that has been optimized to identify communities that are guaranteed to be connected. This resulted in clusters of cells that are more coherent with the biological phenotype and more reliably identify cell populations. The resolution parameter value was optimized by surveying the stability of the resulting clusters, choosing a final value of 0.6. This resulted in the identification of 12 clusters. Cluster annotation in cell populations was obtained by a combination of the following approaches: (1) Scanpy’s rank_genes_groups to identify the most characterizing genes per clusters, (2) gene ontology enrichment analysis performed on the top-50 markers of each cluster by GProfiler^104^ and (3) visualization of specific markers for each neurodevelopmental population. The differential expression analysis was carried on the cells identified as mature neurons (namely, clusters ‘N1’, ‘N2’, ‘N3’) with Wilcoxon rank-sum test (as implemented in Scanpy’s rank_genes_groups) by comparing the different organoid conditions: Toxo-Mecp2 vs CNT, Toxo-HA vs CNT and Toxo-Mecp2 vs Toxo-HA. The differentially expressed genes (DEGs) were then ranked according to the formula $-{\rm{{lo}{g}}}_{10}({P}_{{\rm{adj}}})\cdot {\rm{sign}}(C)$ where ${P}_{{\rm{adj}}}$ and $C$ are the adjusted *P* value (Benjamini–Hochberg correction) and the fold-change of the differential analysis. The ranked DEGs were used to identify the enrichment of a set of gene lists of functional significance obtained from the REACTOME^105^ and KEGG^106^ databases, filtering out all the pathways with more than 100 genes. Three different tests were realized: (1) an over-representation analysis (ORA) by a one-tailed Fisher’s exact test to test for the enrichment of the gene lists in the top 500 DEGs, (2) a similar ORA to test for the enrichment of the gene lists in the bottom 500 DEGs and (3) a gene set enrichment analysis (GSEA)^107^ on the full list of DEGs. The decoupleR Python library^108^ was used to perform both the ORA and the GSEA. Further, the most interesting gene list identified (namely, REACTOME ‘Transcriptional regulation by MECP2’) was used to compute a pathway gene score with the help of Scanpy’s score_genes function. The ‘Reactome transcriptional regulation by MECP2’ gene list includes: *MECP2*, *MOBP*, *SGK1*, *RBFOX1*, *CREB1*, *MEF2C*, *PPARG*, *MIR137*, *GAMT*, *PVALB*, *MIR132*, *IRAK1*, *BDNF*, *CRH*, *SST*, *DLL1*, *GAD2*, *GAD1*, *PTPN1, NOTCH1*, *MET*, *OPRK1*, *PTPN4*, *GPRIN1*, *TRPC3*, *OPRM1*, *GRIN2B*, *SLC2A3*, *GRIN2A*, *GRIA2*, *FKBP5* and *PTEN*. Beyond this main analysis, dimensionality reduction by UMAP and cluster identification were also performed in an alternative workflow, considering both human and *T. gondii* genes. In this case, among the identified clusters, one was specifically composed of *T. gondii*-infected cells. The results of all the differential expression and functional analyses are available in GitHub^100^.

### Molecular cloning

For the plasmids encoding for toxofilin-fused proteins, we used as a backbone a pGRA vector containing the toxofilin cDNA fused to Cre recombinase surrounded by the 1.1 kb genomic sequence upstream of *toxofilin* (‘toxofilin promoter’) and the 3’UTR of *GRA2* (refs. ^16,17^). To generate plasmids for the expression of GRA16-fused genes, *GRA16* and its promoter were amplified from RH *T. gondii* genomic DNA. These GRA16 PCR products were used to replace the *toxofilin* promoter and coding sequence in the pGRA-toxofilin plasmid with the GRA16 promoter and coding sequence using NEBuilder assembly. To generate plasmids for the expression of toxofilin-fused and GRA16-fused therapeutic proteins, we used a combination of NEBuilder assembly, restriction-ligation and ‘around-the-horn’^109^ cloning methods. Codon optimization was performed with Genscript, Twist Bioscience, or by manual changing of codons to eliminate repetitive elements, increase sequence complexity, add or remove restriction sites. Recombinant DNA sequences used to generate the heterologous and codon-optimized protein fusions are described in the key resources table, and full DNA sequences are available in Supplementary Table 1. Truncated GRA16 vectors (HA-fused and HA-MeCP2-fused) were generated from the GRA16-HA and GRA16-MECP2opt vectors using the ‘around-the-horn’ method. A toxofilin-Cre plasmid with bleomycin selection was used for the Pru GRA16-MeCP2:TCre and Pru-TCre strains^47^. All plasmids were validated by Sanger sequencing of the entire coding, promoter and 3’UTR regions (primers described in Supplementary Table 1). Plasmid maps were visualized and processed using the Snapgene software (v.2-6, GSL Biotech).

### Tissue preparation, clearing and refractive index matching

Mice were deeply anaesthetized with ketamine and xylazine and transcardially perfused first with ice-cold PBS, followed by a hydrogel monomer mixture of 4% acrylamide, 0.05% bisacrylamide and 1% PFA^110^. Brains were collected and further incubated passively in the hydrogel mixture for 24 h. The hydrogel was degassed and then polymerized at 37 °C for 2.5 h. Samples were extracted from the polymerized gel and cleared in 8% clearing solution (8% w/w sodium dodecyl sulfate in 200 mM boric acid, pH 8.5) at 37 °C for 3 weeks by passive diffusion. Following the completion of the clearing, the samples were rinsed in PBS (4 times for 1 h each time) and then placed in imaging solution^42^. Cleared brains were refractive index (RI) matched to 1.46 with a modified version of the RI matching solution^110^, which includes triethanolamine (tRIMS). tRIMS was made by mixing Histodenz (Sigma, D2158) (100 mg), PBS (75 ml), sodium azide (10% w/v, 500 µl), Tween-20 (75 µl) and triethanolamine (42 ml)^42^.

### Whole-brain imaging and stitching

Whole-brain images were recorded with a custom-made selective plane illumination microscope (mesoSPIM)^111^. SPIM imaging was done after clearing and RI matching. The laser/filter combinations for mesoSPIM imaging were as follows: at 488 nm excitation, a Triple block 488/561/640 nm bandpass filter (BP523/22, BP594/20, BP704/46) was used as emission filter. Transparent whole brains were imaged at a voxel size of 3.26 × 3.26 × 3 µm^3^ (*X* × *Y* × *Z*). Scanning an entire brain required splitting the image into 16 tiles per channel (8 tiles per brain hemisphere). After the acquisition of one hemisphere, the sample was rotated 180° and the other hemisphere was imaged. The tiles were stitched together with the automatic stitching tool TeraStitcher^112^.

### Image processing of 3D whole-brain samples

Cell segmentation and quantification was performed using an in-house-developed algorithm, aimed at high-speed processing of whole-brain mouse datasets. The algorithm consists of three main steps^113^: (1) image restoration, (2) detection of candidate cells and (3) discrimination of non-cell structures. Image restoration was employed to correct imperfections in the intensity distribution of the captured images. We targeted two effects: correcting the uneven distribution of autofluorescence intensity throughout the sample and removing pixel noise, introduced during the digitization of the image via the microscope camera. In the case where the structures of interest were relatively small compared with the length scale of intensity variations in the background, the background intensity gradients could be eliminated with a simple step. Similar to refs. ^113,114^, we modelled the background intensity as a convolution of the raw data with a Gaussian distribution with variance *σ*^2^. *σ* was chosen to be larger than the typical foreground structure radius but smaller than the effective radius of background regions with high autofluorescence. In the analysis, we used *σ* = 10 pixels. The intensity of the raw data is denoted as *I*(*x*, *y*, *z*), hereafter denoted as *I* for simplicity, where *x*, *y*, *z* are the row, column and depth coordinates, respectively. The background intensity is denoted as *I*_*σ*_.

1 $${I}_{\sigma }(x,y,z)=\frac{1}{B}\mathop{\sum}\limits_{i,\;j,k=-w}^{w}I(x+i,y+j,z+k){\rm{exp}}\left(-\frac{{\rm{i}}^{2}+{\rm{j}}^{2}+{\rm{k}}^{2}}{2{{\sigma}_{\rm{n}}}^{2}}\right)$$

with *B* being a normalization factor such that the Gaussian distribution within a cube with side 2*w* + 1 integrates to 1. The background undulations were removed by dividing the raw data with the smoothed data^115^, giving the normalized data *I*_n_.

2 $${I}_{\rm{n}}=\frac{I}{{I}_{\sigma }}$$

In the normalized data, pixels corresponding to the background have values around 1, while the foreground structures are expected to have values much greater than 1. This facilitates the separation between foreground and background pixels. To suppress the digitization noise, we applied a convolution with a Gaussian kernel to the normalized image to obtain the denoised image *I*_d_.

Cell detection was performed via edge-detection methods. We used the Laplacian of Gaussian (LoG) to detect closed surfaces of local intensity maxima. The output of the LoG operation is denoted as *I*_LoG_.

3 $${I}_{{\rm{LoG}}}=-{\nabla }^{2}{I}_{\rm{d}}=-\left(\frac{{\partial }^{2}{I}_{\rm{d}}}{{\partial x}^{2}}+\frac{{\partial }^{2}{I}_{\rm{d}}}{{\partial y}^{2}}+\frac{{\partial }^{2}{I}_{\rm{d}}}{{\partial z}^{2}}\right)$$

At locations of sharp intensity changes, *I*_LoG_ is positive inside local maxima (that is, the cell interior), negative in between neighbouring local maxima (that is, the cell exterior) and zero along the edge separating the internal/external regions. All regions with *I*_LoG_ > *α* are thus considered potential cells, where 0 < *α* << 1 is a small numerical threshold used to exclude artefacts generated by the LoG operation on background noise. The spatial derivatives in the Laplacian were approximated via the central finite difference scheme in 3D.

To exclude artefacts and non-cell structures, we made a set of assumptions about the intensity and shape of the true cells: (1) the target cells are expected to have a high intensity relative to their surrounding background, thus having *I*_d_ much larger than 1, and (2) a spherical-like shape. The image restoration step conveniently allowed setting a minimum threshold *I*_d,min_ on the maximum object intensity for an object to be accepted as a true cell. We set *I*_d,min_ = 1.1 and excluded all objects not meeting this threshold from further analysis. For all remaining objects, we computed a set of features characterizing their morphology: asphericity, acylindricity, relative shape anisotropy, radius of gyration *R*_g_, ratio of object volume to volume of sphere with radius *R*_g_, and the eigenvalues of the gyration tensor. Combined with the averaged denoised intensity and maximum denoised intensity per object, these features were then used to train a machine learning algorithm, namely, a random forest classifier (RFC) to classify objects in two categories: cells and non-cells. In our datasets, objects belonging to the first category included cells and cell conglomerates. Objects belonging to the latter include single vessels as well as larger parts of connected vasculature. The training of the RFC was performed using 1,700 manually labelled objects from sample 4864. In a setting of 80%:20% random split of the labels in training and testing datasets, the RFC achieved on average 98% prediction accuracy in the training dataset and 90% in the testing dataset. The trained RFC was then used for the classification of detected objects into cells and non-cells in all mouse brain samples. A representative output from the cell detection pipeline is shown in Extended Data Fig. 6. Detected cells were aligned on the Allen Brain Atlas^43^ with pixel resolution of 25 µm, using the elastix image registration software^116^ for the comparison of data among different samples. A representative output of the alignment process is shown in Extended Data Fig. 6.

The segmentation accuracy was validated against manual annotations of a chunk from sample 4864 generated by a domain expert. Sample 4864 was chosen as it contained the highest number of cells. Annotations were generated in a chunk of size 200 × 200 × 40 pixels, containing 442 cells. In the annotated data, a pixel was assigned value 1 if it corresponded to a cell and value 0 otherwise. The segmentation algorithm was run on the same chunk and its predictions were compared to the manual annotations. Results are shown in Extended Data Fig. 6. In computing the validation statistics, we took into account that the boundary separating cells and background is uncertain and depends on the choice of image contrast settings. Therefore, if the core of a cell was correctly detected by the algorithm, any mismatch of its boundary with respect to the manual annotations was ignored. Under this premise, the volume of true positive (TP) pixels was 98.4% of the total true cell volume and the volume of true negative (TN) pixels was 99.5% of the total true non-cell volume. A convergence of TP and TN percentages with respect to the number of *z*-slices included in the computation is shown in Extended Data Fig. 6. In addition, an overlay of the raw and segmented data is shown in Extended Data Fig. 6. The pipeline for image processing of 3D whole-brain samples is available via GitHub^117^.

### Statistical analysis of 3D whole brains

The quantification of spatial cell distribution was performed using density plots depicting the volume of detected cells inside a cubic voxel with side 25 µm (Allen Brain Atlas resolution). Coronal sections of the cell volume distribution were overlayed on the respective slices of the reference Allen Brain Atlas for every sample (Extended Data Fig. 6). For every group, the average cell volume distribution was computed by taking the mean over the samples belonging to each group. Using the annotated Allen Brain Atlas, we identified in which brain region each cell resides and computed the total cell volume detected in five brain regions: brainstem, hippocampus, hypothalamus, cortex and thalamus (Extended Data Fig. 6). The cerebellum was removed from the final analysis because it displayed high levels of autofluorescence from Purkinje cells (detected cells in the negative control and infected brains in Extended Data Fig. 6). Low levels of ZsGreen activation were observed in several loci in mice treated with saline, which were clustered and consistent with background spontaneous ZsGreen expression commonly observed in Ai6 mice^44^. The group-wise average cell volume and corresponding standard deviation of each brain region was then computed by taking into account all processed samples from each group. The code used for the statistical analysis of 3D whole brains is available in GitHub^117^.

### Plaque assays

Parasites were mechanically egressed and 500 tachyzoites were seeded into 6-well plates containing confluent host cell monolayers. Media were replaced with DMEM (11965) supplemented with 10% or 1% FBS and plates were left to culture for 7 days in a 37 °C humidified 5% CO_2_ incubator. Plate contents were fixed with ice-cold 100% methanol and then stained with crystal violet solution for 2 h. Images were taken using a Google Pixel 3A phone and plaque area measured using the Fiji distribution of ImageJ^118^. The numbers of plaques for each well’s contents were counted within the perimeters of two pre-selected areas with diameters of 14 mm. Statistical analysis was conducted using GraphPad Prism v.7.0 for Windows.

### Parasite replication assay

Parasites were seeded onto 6 cm dishes containing HFF lawns and grown in either DMEM (11965) supplemented with 10% FBS or 1% FBS, or glucose-deficient DMEM (11966) for 3 days. Plates containing glass coverslips coated in HFF lawns were pre-treated with respective culturing media conditions for >1 day. Parasites were then mechanically egressed and seeded onto the coverslips and incubated for 1 h in a 37 °C humidified 5% CO_2_ incubator. Following incubation, well contents were gently washed to remove extracellular parasites, and media replaced before being returned to the incubator and cultured for 24 h. After 24 h, plate contents were fixed in 4% PFA. Cells were permeabilized and blocked in 0.2% Triton X-100 2% bovine serum albumin in PBS for 20 min at r.t. and then incubated with α-IMC1 (1:1,000, gift from Prof. Dominique Soldati-Favre) primary antibody for 1 h at r.t. Plate contents were washed with 0.2% Triton X-100 in PBS and cells were then incubated with Alexa Fluor goat anti-rat 488 (A-11006, Invitrogen, 1:1,000) for 1 h at r.t. Coverslips were mounted on glass microscopy slides with Fluoromount-G containing DAPI (Southern Biotech). Statistical analysis was conducted using GraphPad Prism v.7.0 for Windows.

### Red-green invasion assay

Parasites were mechanically egressed, seeded onto a plate containing coverslips coated in HFF lawns and left for 30 min in a 37 °C humidified 5% CO_2_ incubator. Plate contents were then gently washed with PBS and fixed in 4% PFA. Cells were blocked in 2% bovine serum albumin in PBS for 20 min at r.t. and then incubated with α-SAG1 (1:400, gift from Dr David Smith) primary antibody for 1 h at r.t. Plate contents were washed with PBS and cells were then incubated with Alexa Fluor goat anti-rat 594 (A-11007, Invitrogen, 1:1,000) for 1 h at r.t. Plate contents were then subjected to the same permeabilization, blocking and staining methods as described in the parasite replication assay. Statistical analysis was conducted using GraphPad Prism v.7.0 for Windows.

### Reporting summary

Further information on research design is available in the Nature Portfolio Reporting Summary linked to this article.

### Supplementary information

Reporting Summary

Supplementary Table 1 Oligonucleotide sequences used in this study, recombinant DNA used in this study and proteins surveyed as candidates for delivery by *T. gondii* as therapeutic targets for human neurological conditions.

### Source data

Source Data Figs. 2 and 3, and Extended Data Fig. 1 Raw data for the graphs presented.

Source Data Fig. 4 and Extended Data Fig. 7 Raw uncropped blot for Fig. 4c and gels for Extended Data Fig. 7b.

## Figures and Tables

**Extended Data Table 1:**
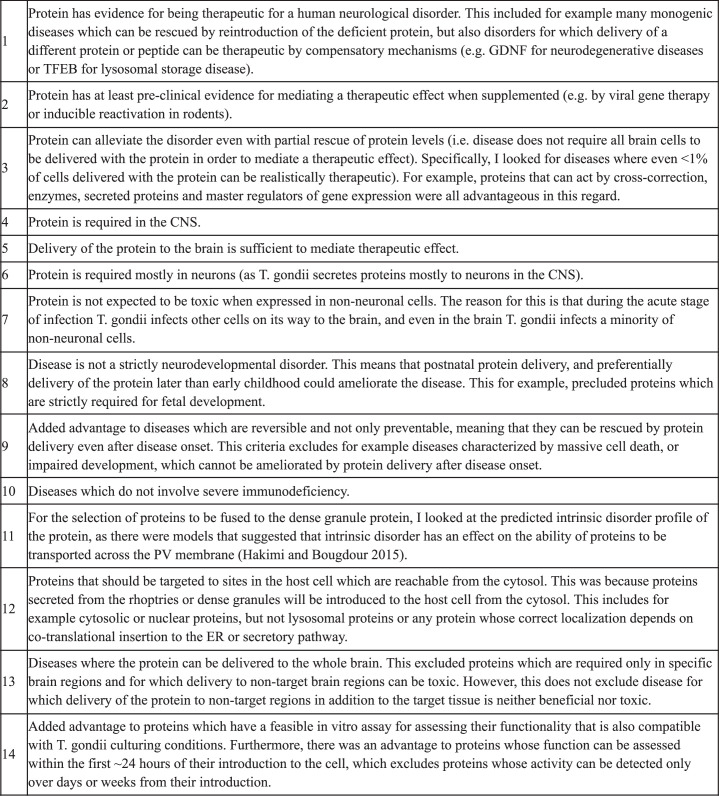
Criteria for selection of candidate therapeutic proteins for delivery by T. gondii

**Extended Data Table 2:**
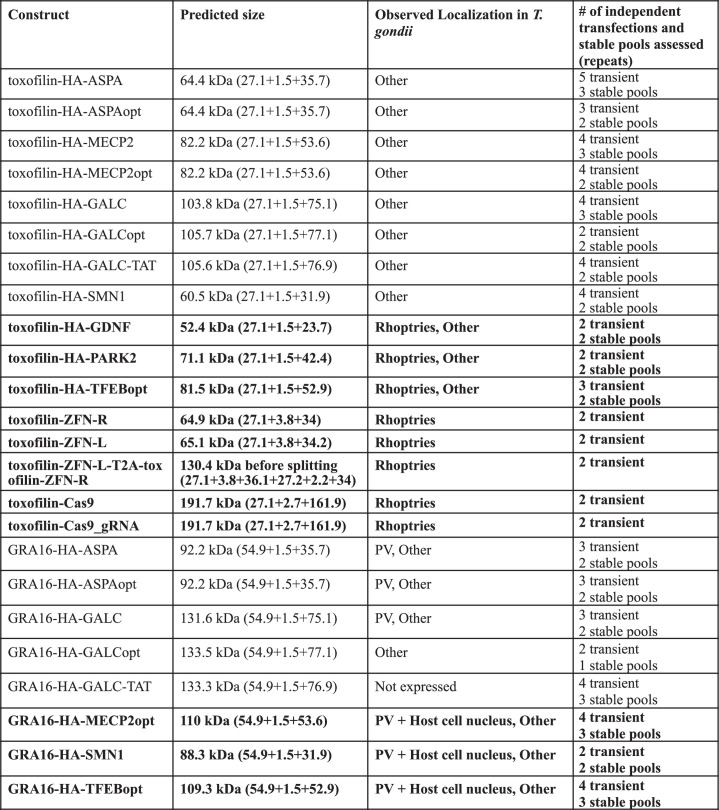
New toxofilin and GRA16 fusion constructs tested in this study

**Extended Data Table 3:**
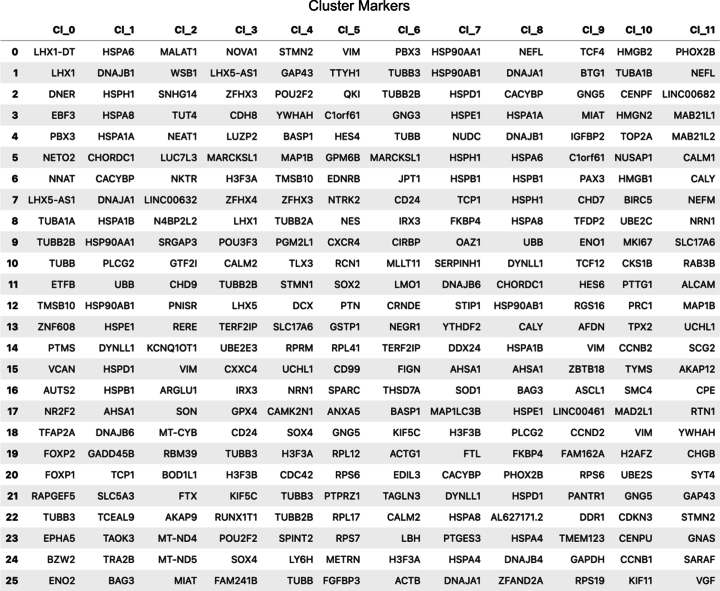
Top gene markers characterizing the Leiden clusters from the organoid single-cell sequencing analysis

Summary table of the first 25 gene markers (computed with scanpy.tl.rank_genes_groups function in Scanpy) characterizing each of the Leiden clusters shown in Extended Data Fig. 3 panel D.

## Data Availability

DNA sequences and raw data used for the graphs and for statistical analyses are available in the manuscript source data and supplementary materials. The single-cell transcriptomic sequencing data are available at: https://www.ncbi.nlm.nih.gov/bioproject/PRJNA934842. The 3D whole-brain image data used for the quantification and statistical analysis in Fig. 6, Extended Data Fig. 5 and 6, as well as video rendering of the 3D imaging data are available via Zenodo at 10.5281/zenodo.10835741 (ref. ^119^) and GitHub at https://github.com/aecon/toxoplasma3D (ref. ^117^). Other raw imaging data and metadata are available in GitHub at https://github.com/shaharbr/Bracha_et_al_2024 (ref. ^94^). Source data are provided with this paper.

## Code availability

Custom code generated during this study is available in GitHub at https://github.com/shaharbr/Bracha_et_al_2024 (ref. ^94^), https://github.com/GiuseppeTestaLab/toxo-organoids (ref. ^100^) and https://github.com/aecon/toxoplasma3D (ref. ^117^).
