# Supplementary material for: Engineering Toxoplasma gondii secretion systems for intracellular delivery of multiple large therapeutic proteins to neurons
Source: Nat Microbiol. 2024 Jul 29;9(8):2051–72. doi: 10.1038/s41564-024-01750-6 (PMC11306108; doi:10.1038/s41564-024-01750-6)
Supplement: Supplementary file 1 — Reporting Summary [file 41564_2024_1750_MOESM1_ESM.pdf]

nature portfolio

Corresponding author(s): Shahar Bracha

Last updated by author(s): May 16, 2024

Reporting Summary

Nature Portfolio wishes to improve the reproducibility of the work that we publish. This form provides structure for consistency and transparency in reporting. For further information on Nature Portfolio policies, see our Editorial Policies and the Editorial Policy Checklist.

Statistics

For all statistical analyses, confirm that the following items are present in the figure legend, table legend, main text, or Methods section.

n/a	Confirmed	
☐	☒	The exact sample size (n) for each experimental group/condition, given as a discrete number and unit of measurement
☐	☒	A statement on whether measurements were taken from distinct samples or whether the same sample was measured repeatedly
☐	☒	The statistical test(s) used AND whether they are one- or two-sided<br>*Only common tests should be described solely by name; describe more complex techniques in the Methods section.*
☐	☒	A description of all covariates tested
☐	☒	A description of any assumptions or corrections, such as tests of normality and adjustment for multiple comparisons
☐	☒	A full description of the statistical parameters including central tendency (e.g. means) or other basic estimates (e.g. regression coefficient) AND variation (e.g. standard deviation) or associated estimates of uncertainty (e.g. confidence intervals)
☐	☒	For null hypothesis testing, the test statistic (e.g. F, t, r) with confidence intervals, effect sizes, degrees of freedom and P value noted<br>*Give P values as exact values whenever suitable.*
☒	☐	For Bayesian analysis, information on the choice of priors and Markov chain Monte Carlo settings
☒	☐	For hierarchical and complex designs, identification of the appropriate level for tests and full reporting of outcomes
☒	☐	Estimates of effect sizes (e.g. Cohen's d, Pearson's r), indicating how they were calculated

Our web collection on statistics for biologists contains articles on many of the points above.

Software and code

Policy information about availability of computer code

Data collection	No specialized software was used for data collection
Data analysis	Software used to analyze data presented in this study include: Python 3.7, Prism 7.0 GraphPad, CellProfiler v3.1.5 (Carpenter et al. 2006), SnapGene versions 2-6 (GSL biotech), ToxoDB tools (Gajria et al. 2007), Fiji distribution of ImageJ version 2.0 (Schindelin et al. 2012), OME bio-formats plugin for ImageJ (Goldberg et al. 2005), Diffraction PSF 3D plugin for ImageJ (Dougherty 2005), Iterative Deconvolution 3D plugin for ImageJ (Dougherty 2005), IUPred2A (Mészáros, Erdos, and Dosztányi 2018), PrimerBLAST NCBI software (Ye et al., 2012), CHOPCHOP v3 (Labun et al. 2019), elastix image registration software (Klein et al., 2010), ITK-SNAP (Yushkevich et al 2006), CellRanger software v6.1.2 and v7.0.0, Leica LAS AF software v. 2.7.3.9723.<br><br>In addition, the high content imaging, single-cell transcriptomics and 3D brain imaging analyses were performed using the custom code available at: https://github.com/shaharbr/Bracha_et_al_2024, https://github.com/GiuseppeTestaLab/toxo-organoids and https://github.com/aecon/toxoplasma3D.

For manuscripts utilizing custom algorithms or software that are central to the research but not yet described in published literature, software must be made available to editors and reviewers. We strongly encourage code deposition in a community repository (e.g. GitHub). See the Nature Portfolio guidelines for submitting code & software for further information.

Data

Policy information about availability of data

All manuscripts must include a data availability statement. This statement should provide the following information, where applicable:

- Accession codes, unique identifiers, or web links for publicly available datasets
- A description of any restrictions on data availability
- For clinical datasets or third party data, please ensure that the statement adheres to our policy

Reference genomes used for sequencing data analysis include the human genome GRCh38 distributed with cellranger (release 2020-A) and the Toxoplasma gondii ME49 genome (release 52) from ToxoDB (ToxoDB.org). The imaging analysis of the cleared brains used the Allen Brain Atlas (Allen Institute for Brain Science, 2011). Single cell sequencing analysis used the REACTOME (reactome.org) and KEGG (https://www.genome.jp/kegg/pathway.html) databases for functional enrichment analysis. DNA sequences and raw data used for the graphs and for statistical analyses are available in the manuscript source data and supplemental materials. The single cell transcriptomic sequencing data is available at: https://www.ncbi.nlm.nih.gov/bioproject/PRJNA934842 . The 3D whole-brain image data used for the quantification and statistical analysis in Fig. 6, Extended Data Fig. 5 and 6 as well as video rendering of the 3D imaging data are available on: https://zenodo.org/doi/10.5281/zenodo.10835741 and https://github.com/aecon/toxoplasma3D . Other raw imaging data and metadata are available on: https://github.com/shaharbr/Bracha_et_al_2024.

Research involving human participants, their data, or biological material

Policy information about studies with human participants or human data. See also policy information about sex, gender (identity/presentation), and sexual orientation and race, ethnicity and racism.

Reporting on sex and gender	NA
Reporting on race, ethnicity, or other socially relevant groupings	NA
Population characteristics	NA
Recruitment	NA
Ethics oversight	NA

Note that full information on the approval of the study protocol must also be provided in the manuscript.

Field-specific reporting

Please select the one below that is the best fit for your research. If you are not sure, read the appropriate sections before making your selection.

☒ Life sciences ☐ Behavioural & social sciences ☐ Ecological, evolutionary & environmental sciences

For a reference copy of the document with all sections, see nature.com/documents/nr-reporting-summary-flat.pdf

Life sciences study design

All studies must disclose on these points even when the disclosure is negative.

Sample size

Sample sizes for the mouse brain imaging experiments was determined in consultation with the groups of Prof. Adriano Aguzzi, Prof. Oded Rechavi and Prof. Anita Koshy. The sample size chosen, ranging from a minimum of 6 to a maximum of 10, was informed by previous data and experimental statistics from Aguzzi's laboratory (Kirschenbaum et al, 2022). For negative control saline-injected samples, a smaller number of samples was considered sufficient because of the reduced variability in this condition.
Fig 3A-D (cell culture): N=3 (i.e., 3 individual neuronal dissections and infections were done), 50 FOV. N=3 is a generally accepted minimum of independent experiments confirm that the findings are reproducible. 50 FOVs has been confirmed by us in previous experiments as more than enough to capture the measured statistical values robustly.
Fig 5A-B: N=12 was chosen based on pilot study to determine the timing compatible with capturing the effects of GRA16-MeCP2. Since the pilot study suggested catching such signaling would be rare- we used a high N (12).
Fig 5D:-given the high number of mice without cysts (Fig 5B) and the analysis this would require (looking at all neurons in multiple FOV for HA staining), we decided to pursue doing such an analysis in a subset of mice that had the highest level of cyst burden based on 5B. We chose the top 3, as it is generally accepted minimum of independent experiments to confirm that the findings are reproducible.
Fig 5E-M: As inflammation from T. gondii infection is known to be stereotypical in all infected mice (Strack et al., 2002; Brenier-Pinchart et al. 2004; Stenzel et al., 2004; Wilson et al. 2005; Robben et al., 2005; Benevides et al., 2008; Drögemüller et al., 2008; Ploix et al., 2011; Cekanaviciute et al., 2014; Biswas et al., 2015; Hidano et al. 2016, McGovern et al. 2020), a smaller N=5 was selected to comply with the protocol's ethical guidelines and avoid using more animals than necessary. We have also validated that this number of mice enables detection of inflammatory changes vs. saline in previous paper (Cekanaviciute J Immunology 2014; Cabral, Tuladhar et al Plos Path 2016; Cabral ASN Neuro 2017; Tuladhar et al Plos Path 2019; Merritt, Johnson mSphere 2020; Merritt Plos One 2024). Based on empirically derived standard deviations, N =5 for infected mice and 2-3 for saline allows us to detect ≥30% change with power of 80%, alpha = 0.05.

Fig 6: N=7-8 mice/infected group was based upon our prior work (Mendez et al 2019) in counting GFP-expressing neurons in infected mice. Based on this work, we derived how many mice would be useful for toxofilin-Cre and then used about the same number for GRA16-Cre. Extended Data Figure 7: N=10 for the plaque area analysis was determined based on a power analysis. Sample size and methodology for the replication assay was chosen to align with the following papers: Xia, et al 2018, Oppenheim et al 2014. Sample size and methodology for the red-green invasion assay was chosen to align with the following papers: Huynh et al 2003, Huynh et al 2006, Stasic et al 2019, Sparvoli et al 2022.

Data exclusions	In the analysis of 3D brain distribution of the secreted proteins, the cerebellum was removed from the final analysis because the cerebellum displayed high levels of autofluorescence from Purkinje cells (detected cells in the negative control and infected brains in Extended Data Figure 6).<br>In the high-content imaging analysis of T. gondii infection and protein secretion, the data processing involved removal of images in which parasite identification failed (infected wells with no identified vacuoles or more than >400 identified vacuole), removal of wells with extreme outlier fluorescence intensity (>5 SD from mean of condition) and removal of wells with outlier numbers of identified parasitophorous vacuoles (>3 std from mean of condition). In addition, although we initially recorded timepoints above 24 hours post-inoculation, we removed from the dataset as we found that the parasite vacuoles were too large for efficient segmentation and host cell association by CellProfiler.
Replication	Assessment of the localization of all the Toxofilin and GRA16 fusions were done over 2-5 independent repeats of transfection, staining and imaging, as detailed in Extended Data Table 2. All the experiments in mice were done with 5-12 mice per group, and any imaging-based assay was performed on many cells, FOV and/or tissue sections, as detailed in the figure legends. All the experiments on the effects of the LDH mutations were performed over 3 replicates. The experiments with the organoids were performed on 3 replicates from each condition.
Randomization	The age and gender of the mice were balanced among the different the experimental groups:<br>The infection experiments at 18 dpi were performed in 36 mice aged 4-5 months (6 female and 6 male in each group).<br>The infection experiments at 1 and 3 mpi were performed in 33 mice aged 3-5 months (3-5 females and 5-7 males in each group).<br>The infection experiments for the 3D brain distribution characterization were performed in 22 mice aged 4-6 months (5-6 females and 4-5 males per group with 2 saline controls).<br>Other than this, all variables besides the tested conditions were randomized among the different experimental conditions.
Blinding	Investigators quantifying CD3+ and Iba1+ cells were blinded to the infection status of the mouse until after the counts were completed.<br>For the scoring of tissue inflammation, the degree of inflammation for each tissue type was assessed as follows: 0 (no inflammation), 1 (moderate inflammation), and 2 (high inflammation). 8 FOV per tissue type (heart/liver/lungs) per mouse were imaged and analyzed. For each tissue type, scores of the individual FOV/mouse were averaged to yield an average inflammation score per mouse. Investigators assigning inflammation scores for the tissue samples were blinded to infection status of the mouse until after data were collected.<br>Synaptophysin-TdTomato punctae were quantified in a double-blinded manner and repeated by two independent assessors.<br>The percentage of diffuse vs punctate HA staining in neurons was quantified in a double blind manner.

Reporting for specific materials, systems and methods

We require information from authors about some types of materials, experimental systems and methods used in many studies. Here, indicate whether each material, system or method listed is relevant to your study. If you are not sure if a list item applies to your research, read the appropriate section before selecting a response.

Materials & experimental systems

n/a	Involved in the study
☐	☒ Antibodies
☐	☒ Eukaryotic cell lines
☒	☐ Palaeontology and archaeology
☐	☒ Animals and other organisms
☒	☐ Clinical data
☒	☐ Dual use research of concern
☒	☐ Plants

Methods

n/a	Involved in the study
☒	☐ ChIP-seq
☒	☐ Flow cytometry
☒	☐ MRI-based neuroimaging

Antibodies

Antibodies used	Rat anti-HA (Sigma-Aldrich, #ROAHAHA), Rabbit anti-HA (Cell Signaling Technology #C29F4), Rabbit anti-T.gondii (Abcam #Ab138698), Mouse anti-TUBB3 (Biolegend #801202), goat anti-SOX2 (R&D system #AF2018), anti-MAP2B (BD Biosciences 610460), mouse anti-NeuN (Merck-Millipore #MAB377), Rabbit anti-IMC1 (gift from Prof. Dominique Soldati-Favre), Rabbit anti-MeCP2 (Cell Signaling, #3456), Mouse anti-NeuN (Abcam #ab104224), Biotin conjugated anti-NeuN (Millipore, #MAB377B), anti-TgSAG1 (gift from Dr David Smith), anti-toxoplasma (Abcam Ab138698), anti-ROP2/4 (gift from Prof. Dominique Soldati-Favre), anti-Iba-1 (Wako Pure Chemical Industries, 019–19741), anti-mouse CD3ε 500A2 (BD Pharmingen, 550277), biotinylated goat anti-rabbit (Vector Laboratories, BA-1000), biotinylated goat anti-hamster (Vector Laboratories, BA-9100), Anti-Rat Goat Secondary Antibody Alexa Fluor® 488 conjugate (Invitrogen, #A-11006), Anti-Rat Goat Secondary Antibody Alexa Fluor® 594 conjugate (Invitrogen #A-11007), Anti-Rabbit Goat Secondary Antibody Alexa Fluor® 488 conjugate (Invitrogen #A-11008), Anti-Rabbit Goat Secondary Antibody Alexa Fluor® 594 conjugate (Invitrogen #A-11012), Anti-Mouse Goat Secondary Antibody Alexa Fluor® 594 conjugate (Invitrogen #A-11005).

Validation	The antibodies were validated by the manufacturers, and also validated using negative and positive controls in our experiments, in this paper and in previous work from our lab and from others.<br>Millipore antibodies manufacturer statement: Routinely evaluated by immunohistochemistry on brain tissue.<br>BD Pharmigen antibodies manufacturer statement: Flow cytometry (Routinely Tested), Immunohistochemistry-frozen (Tested During Development). In addition, we have validated these antibodies in previous papers, confirming that the cell type and morphologies observed are as expected given their targets and our controls: Cekanaviciute J Immunology 2014; Cabral, Tuladhar et al Plos Path 2016; Cabral ASN Neuro 2017; Tuladhar et al Plos Path 2019; Merritt, Johnson mSphere 2020; Merritt Plos One 2024.<br>Wako Pure Chemical anti-Iba-1: in all of our experiments in this paper and previous work, the morphology observed with the staining is consistent with the morphology and distribution of microglia and macrophages and not with other brain cell types like neurons, astrocytes and T cells. It is routinely used in the literature.<br>Sigma anti-HA: Function tested in western blot.<br>Cell Signaling anti-HA: HA-Tag (C29F4) Rabbit mAb detects exogenously expressed proteins containing the HA epitope tag. In our hands, works on controls as expected (staining cells expressing HA-tagged proteins, and not staining cells without it).<br>Abcam anti-Toxoplasma: Validated by the manufacturer for ELISA and ICC/IF. In our hands, it works as expected on controls (no staining for samples without T. gondii and specific staining with the expected T. gondii morphology in samples with T. gondii.<br>Biolegend anti-TUBB3: This antibody is well characterized and highly reactive to neuron specific Class III ß-tubulin (ßIII). TUJ1 does not identify ß-tubulin found in glial cells. TUJ1 recognizes an epitope located within the last 15 C-terminal residues. This product has been verified for IHC-F (Immunohistochemistry - frozen tissue sections) on the NanoString GeoMx® Digital Spatial Profiler.<br>anti-SOX2 (R&D systems) was validated by the manufacturer by detection of Human, Mouse, and Rat SOX2 by Western Blot, Detection of SOX2-regulated Genes and Mouse SOX2 by Chromatin Immunoprecipitation, Detection and morphological analysis of SOX2 in Mouse Cortical Stem Cells, Rat Cortical Stem Cells, Zebrafish, ADLF1 and FAB2 Stem Cell Lines by Immunocytochemistry, Detection of Human SOX2 by Simple Western, Detection of SOX2 in glioblastoma and Human SOX2 in cervical biopsies by Immunohistochemistry, Detection of SOX2 in NTera�2 cells (Positive) & HepG2 cells (Negative) by Immunocytochemistry.<br>anti-MAP2B (BD biosceince): validated by the manufacturer for Western blot (Routinely Tested), Immunofluorescence, Immunohistochemistry (Tested During Development), Immunoprecipitation (Not Recommended). Reactivity tested for Rat (QC Testing), Human, Mouse (Tested in Development).<br>anti-IMC1, anti-SAG1, anti-ROP2/4 - widely used antibodies in the T. gondii community. Validated on controls, not showing staining in negative controls (without T. gondii), showing staining with the expected localization and morphology in T. gondii immunostaining, and binding proteins of the expected size in western blots.

Eukaryotic cell lines

Policy information about cell lines and Sex and Gender in Research

Cell line source(s)	Human Foreskin Fibroblasts (male, ATCC #SCRC-1041), LUHMES WT (female, CRL-2927, ATCC, #CRL-2927), LUHMES MECP2-KO (female, Shah et al. 2016), Mouse N2A neuroblastoma (male, Chang and Prasad 1976), UMILi026-A Human induced pluripotent stem cell (female, Giuseppe Testa group)
Authentication	The cell lines used were not authenticated
Mycoplasma contamination	All the cell lines tested negative for mycoplasma
Commonly misidentified lines<br>(See ICLAC register)	None

Animals and other research organisms

Policy information about studies involving animals; ARRIVE guidelines recommended for reporting animal research, and Sex and Gender in Research

Laboratory animals	The infection experiments at 18 dpi were performed in 36 mice aged 4-5 months (6 female and 6 male in each group) of the strain Ai6 RCL-ZsGreen (Jackson Laboratories stock #007906)<br>The infection experiments at 1 and 3 mpi were performed in 33 mice aged 3-5 months (3-5 females and 5-7 males in each group) of the strain Ai6 RCL-ZsGreen (Jackson Laboratories stock #007906).<br>The infection experiments for the 3D brain distribution characterization were performed in 22 mice aged 4-6 months (5-6 females and 4-5 males per group with 2 saline controls) of the strain Ai6 RCL-ZsGreen (Jackson Laboratories stock #007906).<br>The infection experiments for the I.V. administration biodistribution were performed in 10 mice aged 4 weeks (5 males per group) of the strain C57BL/6J (Envigo RMS Israel stock).<br>The synaptophysin-TdTomato primary neuronal cultures were generated from E17 embryos of mice from the strain Ai34(RCL-Syp/tdT)-D (Jackson Laboratories stock #012570).<br><br>For all experiments besides Extended Data Figure 4J, mice were kept and bred at the University of Arizona Animal Care facilities. All mice were housed in specific-pathogen-free University of Arizona Animal Care facilities in the following conditions: 14 hr/10 hr light/dark cycle, ambient temperature between 20-24°C, and humidity of 30-70%. For Extended Data Figure 4J, mice were housed at the CRO MD Biosciences in Ness Ziona, Israel in the following conditions: 12 hr/12 hr light/dark cycle, ambient temperature between 17-23°C, and humidity of 30-70%.
Wild animals	The study did not involve wild animals

Reporting on sex	In all the experiments performed in mice besides Extended Data Figure 4J, we balanced as much as possible the number of females and males in each condition, as detailed above. In our data, the results from the female and male mice are aggregated, in accordance with the standards in the field. For the IV biodistribution experiment in Extended Data Figure 4J, all mice used were males in order to reduce variance.
Field-collected samples	The sample did not involve samples collected from the field.
Ethics oversight	For all the experiments performed in mice besides Extended Data Figure 4J, the mice protocols used in this study were approved by the University of Arizona Institutional Animal Care and Use Committee (#A-3248-01, protocol #12–391).<br>For the IV biodistribution experiment in Extended Data Figure 4J, the protocol was approved by the Israeli Committee for Ethical Conduct in the Care and Use of Laboratory Animals.

Note that full information on the approval of the study protocol must also be provided in the manuscript.

Plants

Seed stocks	NA
Novel plant genotypes	NA
Authentication	NA
