# Supplementary figures and images for: Engineering Toxoplasma gondii secretion systems for intracellular delivery of multiple large therapeutic proteins to neurons
Source: Nat Microbiol. 2024 Jul 29;9(8):2051–72. doi: 10.1038/s41564-024-01750-6 (PMC11306108; doi:10.1038/s41564-024-01750-6)

Raw uncropped pull-down blots - Figure 4C

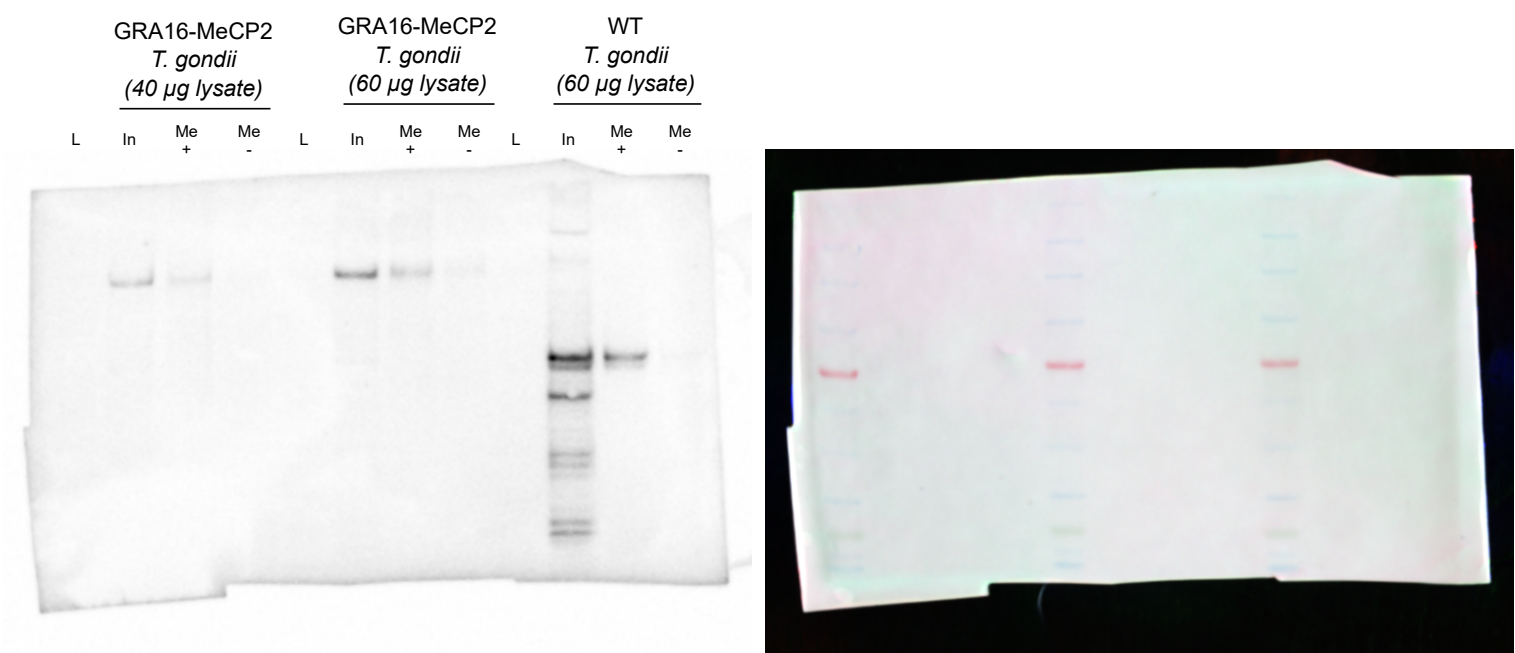

Raw uncropped gel images - Extended Data Fig 7B

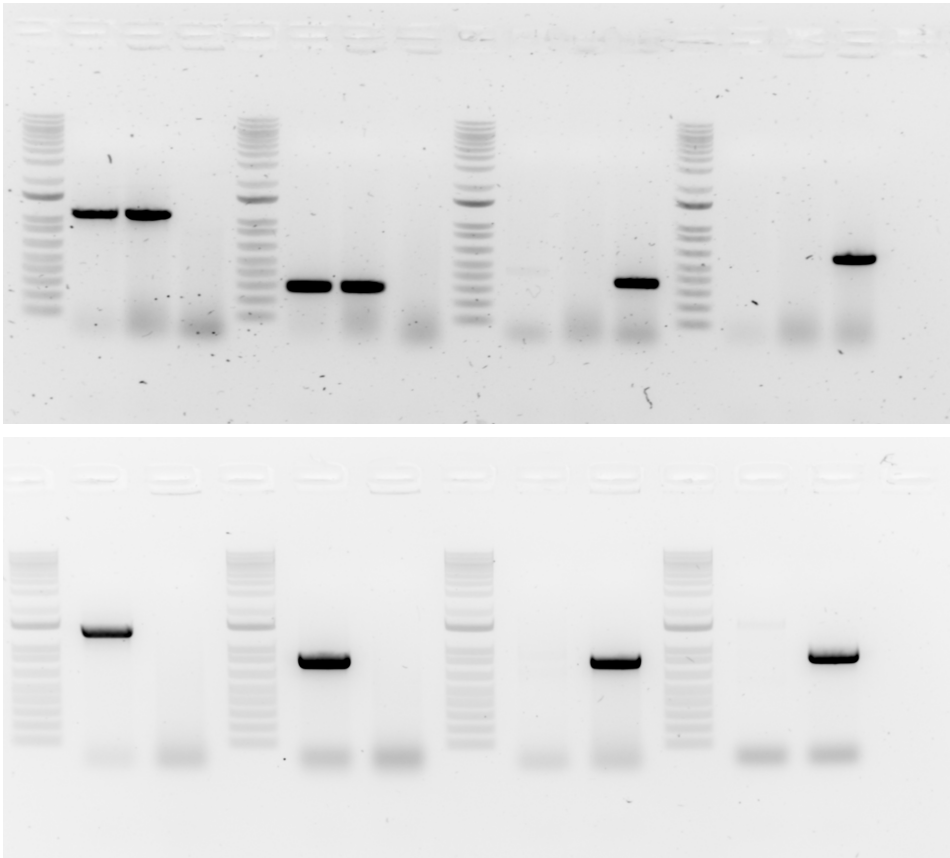

Supplement: Supplementary file 4 — Raw uncropped blot for Fig. 4c and gels for Extended Data Fig. 7b. [file 41564_2024_1750_MOESM4_ESM.pdf]
